# The comprehensive expression and functional analysis of m6A modification “readers” in hepatocellular carcinoma

**DOI:** 10.18632/aging.204217

**Published:** 2022-08-12

**Authors:** Sha Qin, Gaoming Liu, Haoer Jin, Xue Chen, Jiang He, Juxiong Xiao, Yan Qin, Yitao Mao, Luqing Zhao

**Affiliations:** 1 Department of Pathology, Xiangya Hospital, Central South University, Changsha, Hunan, China; 2 Department of Pathology, School of Basic Medical Science, Xiangya School of Medicine, Central South University, Changsha, Hunan, China; 3 Xiangya School of Medicine, Central South University, Changsha, Hunan, China; 4 Department of Radiology, Xiangya Hospital, Central South University, Changsha, Hunan, China; 5 Center for Molecular Medicine, Xiangya Hospital, Central South University, Changsha, Hunan, China; 6 National Clinical Research Center for Geriatric Disorders, Xiangya Hospital, Central South University, Changsha, Hunan, China; 7 Early Clinical Trial Center, Hunan Cancer Hospital and The Affiliated Cancer Hospital of Xiangya School of Medicine, Central South University, Changsha, Hunan, China

**Keywords:** N6-methyladenosine (m6A) modification, hepatocellular carcinoma (HCC), functional analysis, expression profiles, immune cell infiltration

## Abstract

N6-methyladenosine (m6A) modification regulators are essential for the diagnosis and treatment of various cancers. However, the comprehensive analysis about roles of m6A “readers” in hepatocellular carcinoma (HCC) remains unclear. UALCAN, GEPIA2, HPA, Kaplan Meier plotter, cBioPortal, STRING WebGestalt, Metascape and TIMER 2.0 database and Cytoscape software were used to comprehensively analyze the bioinformatic data. We found that m6A “readers” were upregulated at the mRNA level and protein level in HCC patients. Highly expressed YTHDF1, IGF2BP3 and NKAP were positively correlated with advanced HCC stage and had a poor prognosis in OS and PFS. The gene alterations of m6A “readers” happened frequently, and YTHDF3 had the highest mutation rate. The function of m6A “readers” on HCC may be closely correlated with splicing related proteins (including HNRNP family, SNRP family, and SR family), metabolic process, protein binding and RNA splicing related signaling pathways. Moreover, although the correlation of YTHDF3 and CD8+ T cell infiltration, and the correlation of IGF2BP3 and infiltration of mast cells and CAF are negative, most m6A “readers” had a positive correlation with immune cells (including CD8+ T cell, CD4+ T cell, Tregs, B cell, neutrophil, monocyte, macrophage, myeloid dendritic cell, nature killer cell, mast cell, and CAF). Macrophages, CD4+ T cell, Treg, B cell, monocyte, and myeloid dendritic cell had a positively strong correlation (Rho>0.4) with most m6A “readers” (such as YTHDC1, YTHDC2, YTHDF1, IGF2BP3, HNRNPA2B1 and HNRNPC). In conclusion, by comprehensive analysis of m6A “readers”, we found that they were involved in the prognosis of HCC, and m6A “readers” might regulate the development and progression of HCC by participating in some metabolism-related and RNA splicing-related signaling pathways as well as immune cell infiltration.

## INTRODUCTION

Primary liver cancer was reported as the sixth most commonly diagnosed cancer as well as the third leading cause of cancer death worldwide in 2020. Statistical data shows that it was about 906,000 new cases and 830,000 deaths. And liver hepatocellular carcinoma (HCC) accounts for 75% to 85% of primary liver cancer [1]. With the progress of medical technology, the treatment of HCC has been greatly developed. The widespread use of semi-annual surveillance, increasing the proportion of tumors eligible for treatment, and systemic therapies, including emerging immunotherapies, can improve overall survival for HCC patients [2]. Currently, small molecule inhibitors and immunotherapy are emerging as promising treatment options of HCC [3, 4]. With the development of immunotherapy, therapeutic agents are used to target immune cells such as CD4+ CD25+ Foxp3+ regulatory T cells, which led to a huge achievement in HCC treatment [5]. And the biomarker-driven therapies may significantly improve the survival of patients at advanced stages of HCC [6]. Therefore, the identification and combined use of more therapeutic targets and biomarkers may bring new ideas for the treatment of HCC.

N6-methyladenosine (m6A) modification has been a research hot spot in recent years. m6A modification is the most abundant modification in eukaryotic RNA, with roles including maintenance of RNA stability, mRNA precursor splicing, translation initiation, nuclear export, degradation, etc. [7]. It was found that m6A modifications are usually enriched in the stop codon and 3′UTR regions [8]. m6A modifications include m6A “writer”, “eraser” and “reader”, whose functions are to add m6A modifications, remove m6A modifications, and recognize m6A modifications on RNA, respectively. And they were reported to provide some possibilities for the early diagnosis as well as treatment of various cancers [9, 10]. m6A “readers”, which mainly including YTHDC1, YTHDC2, YTHDF1, YTHDF2, YTHDF3, HNRNPA2B1, HNRNPC, NKAP, IGF2BP1, IGF2BP2, and IGF2BP3, are a kind of protein that could recognize the m6A modification in RNA, thus playing roles in RNA metabolism, tumorigenesis, hematopoiesis, viral replication, immune response, and adipogenesis [11]. Nowadays, an increasing number of researches have demonstrated that some m6A “readers” have a potential to predict the prognosis of HCC, such as YTHDF1 [12, 13], and YTHDC2 [14]. However, the comprehensive analysis of m6A “readers” to predict prognosis and diagnosis value of HCC has not been reported.

In this paper, we sort out a comprehensive bioinformatics analysis of the m6A “readers” (including YTHDC1, YTHDC2, YTHDF1, YTHDF2, YTHDF3, IGF2BP1, IGF2BP2, IGF2BP3, HNRNPA2B1, HNRNPC, and NKAP) in HCC. In addition, we discussed their values to be potential therapeutic targets and prognostic biomarkers through various public bioinformatics databases. Furthermore, we selected some key genes from the co-expression genes of m6A “readers”. And these key genes as well as some immune cells infiltration may provide may provide new ideas for the subsequent study of the mechanism of m6A modification in HCC.

## RESULTS

### The up-regulation of the m6A “readers” expression in HCC patients

**Figure 1:**
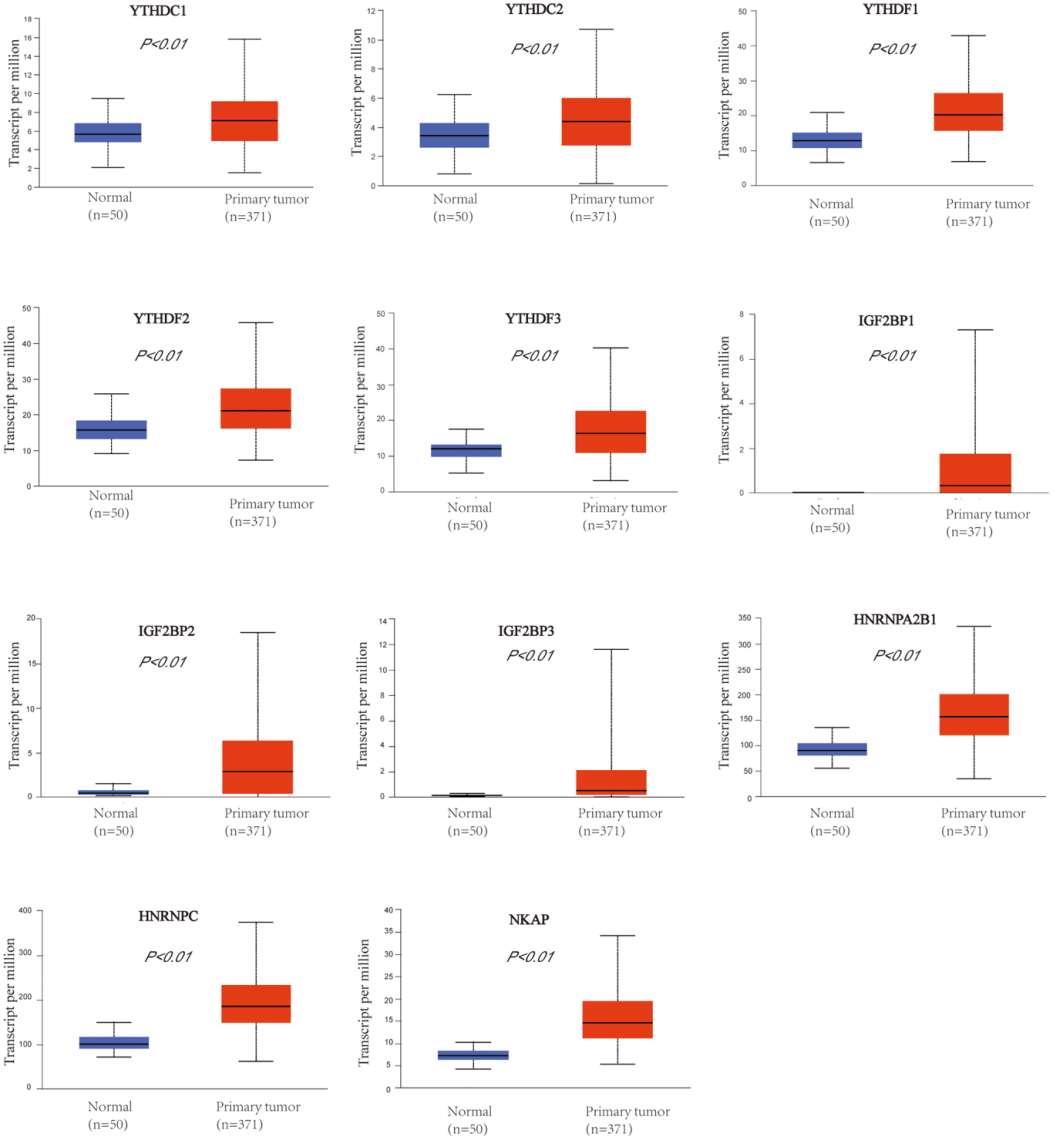
**The mRNA expression profile of m6A “readers” in HCC and normal liver tissues (UALCAN database).** m6A “readers” YTHDC1, YTHDC2, YTHDF1, YTHDF2, YTHDF3, IGF2BP1, IGF2BP2, IGF2BP3, HNRNPA2B1, HNRNPC, and NKAP were expressed significantly higher in HCC than that in normal tissues.

**Figure 2:**
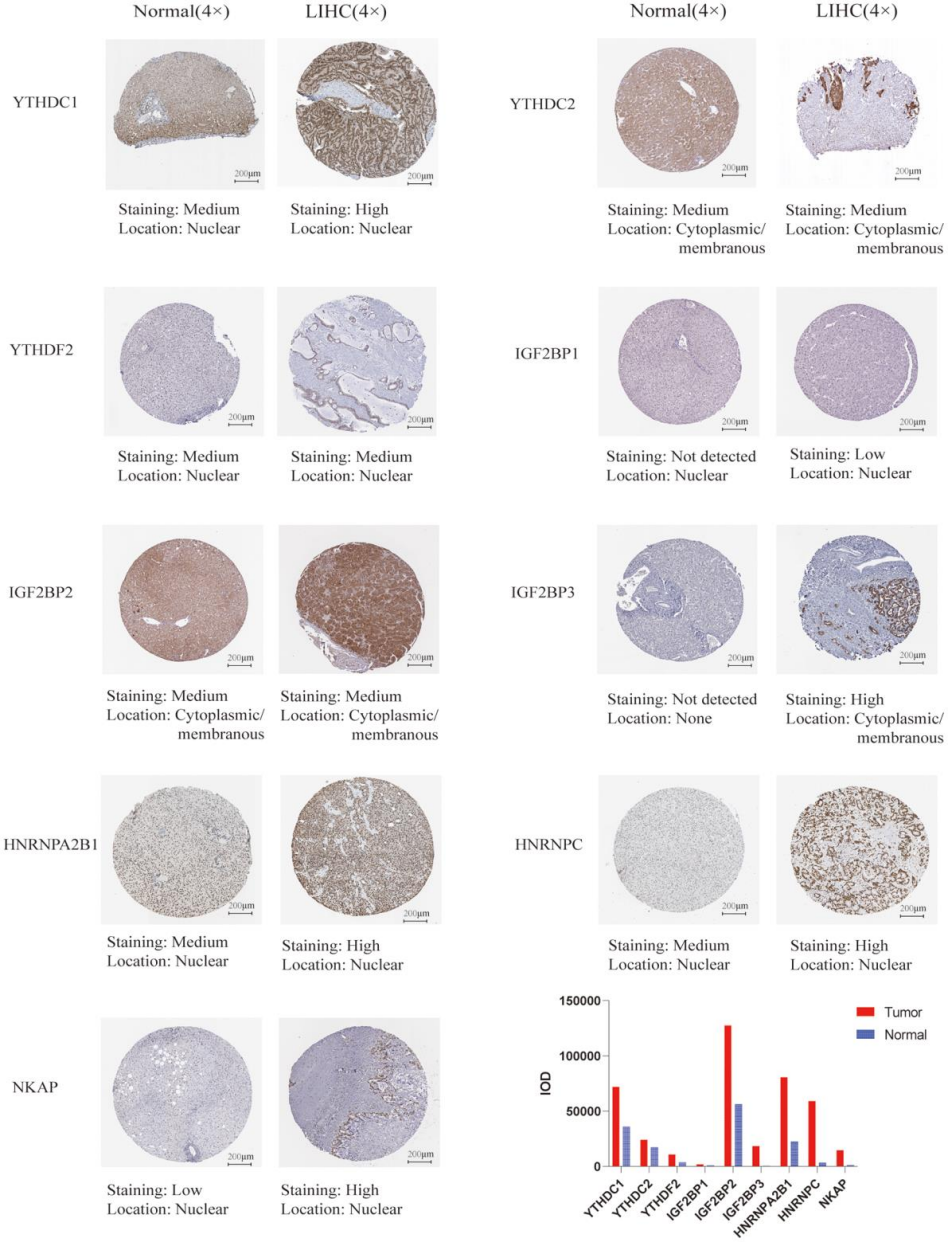
**The protein expression of the m6A “readers” in HCC and normal liver tissues (the Human Protein Atlas database).** The protein expression level of YTHDC1, YTHDC2, IGF2BP1, IGF2BP2, IGF2BP3, HNRNPA2B1, HNRNPC, and NKAP were higher in HCC tissue than in normal liver tissue. In addition, the integrated optical density (IOD) was used to analyze the expression difference between HCC tissues and normal liver tissues.

We used UALCAN database to check the mRNA expression level of m6A “readers” between HCC and normal liver tissue. The expression level of m6A “readers” such as YTHDC1, YTHDC2, YTHDF1, YTHDF2, YTHDF3, IGF2BP1, IGF2BP2, IGF2BP3, HNRNPA2B1, HNRNPC, and NKAP were explored. To our surprise, all of these “readers” expressed significantly higher in HCC than that in normal tissues (Figure 1). Immunohistochemistry could provide us with more information at the tissue level. To determine the differential expression profile of m6A “readers” between normal tissues and HCC., we collected the typical images from the HPA database and analyzed the immunohistochemical staining results by comparing their integrated optical density (IOD) values. (Figure 2). From the Human Protein Atlas (HPA) database, YTHDC1, YTHDF2, IGF2BP1, HNRNPA2B1, HNRNPC and NKAP were detected in live cell nuclear, while YTHDC2, IGF2BP2, IGF2BP3 were expressed in liver cell cytoplasm and membrane. However, the protein expression data of YTHDF1 and YTHDF3 were missed in HPA database. In addition, these stain results showed that the protein expression level of YTHDC1, YTHDC2, YTHDF2, IGF2BP1, IGF2BP2, IGF2BP3, HNRNPA2B1, HNRNPC, and NKAP were higher in HCC tissue than in normal liver tissue. These results were consistent with the differential expression profile of m6A “readers” between normal tissues and HCC (as we showed in Figure 1).

**Figure 3:**
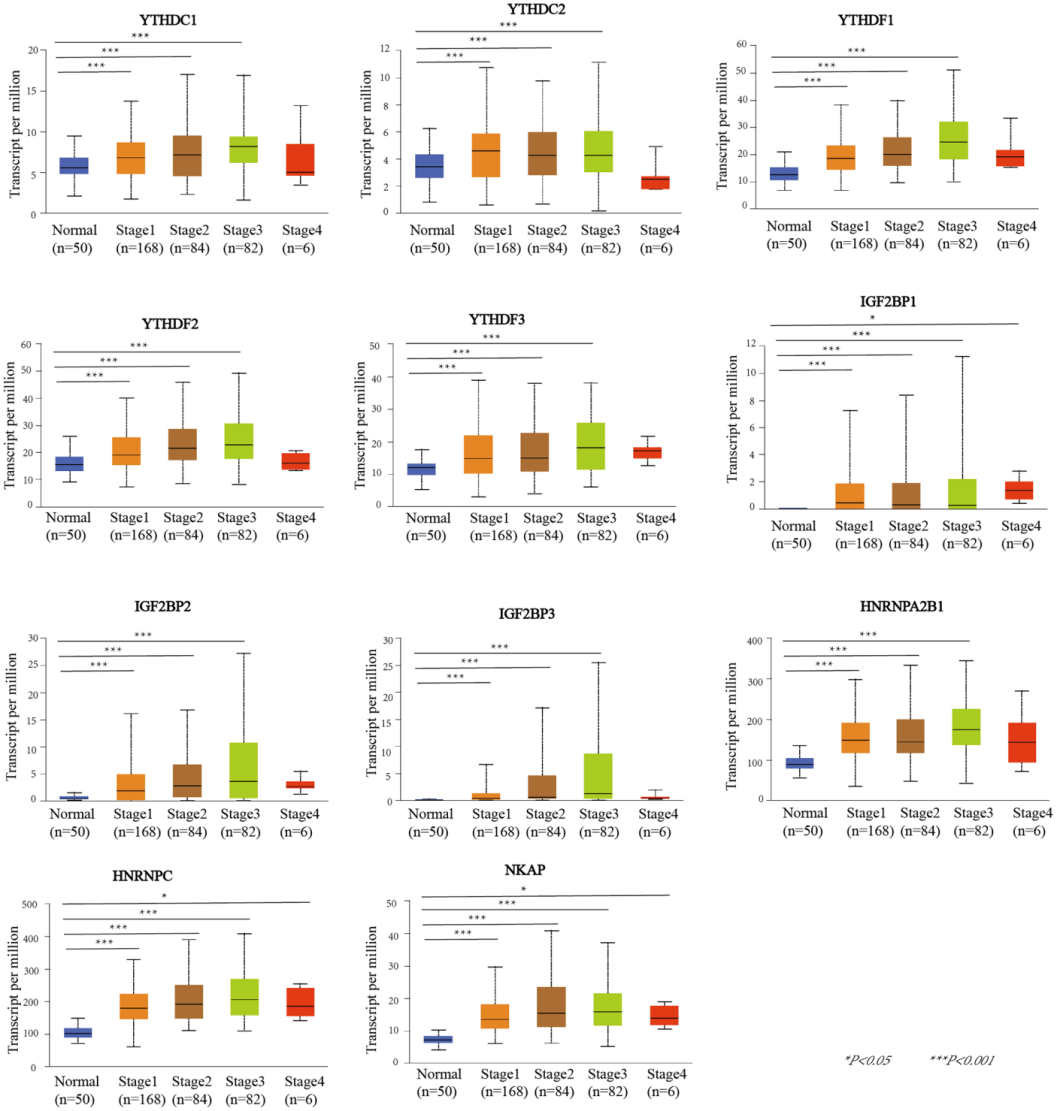
**The relationship between m6A “readers” mRNA expression and pathological stage of HCC patients (UALCAN database).** The mRNA expression level of YTHDC1, YTHDC2, YTHDF1, YTHDF2, YTHDF3, IGF2BP2, IGF2BP3, and HNRNPA2B1 were positively correlated with tumor stage (stage1, stage2, and stage3). The mRNA expression levels of IGF2BP1, HNRNPC and NKAP were positively correlated with tumor stage (stage1, stage2, stage3, and stage4).

**Figure 4:**
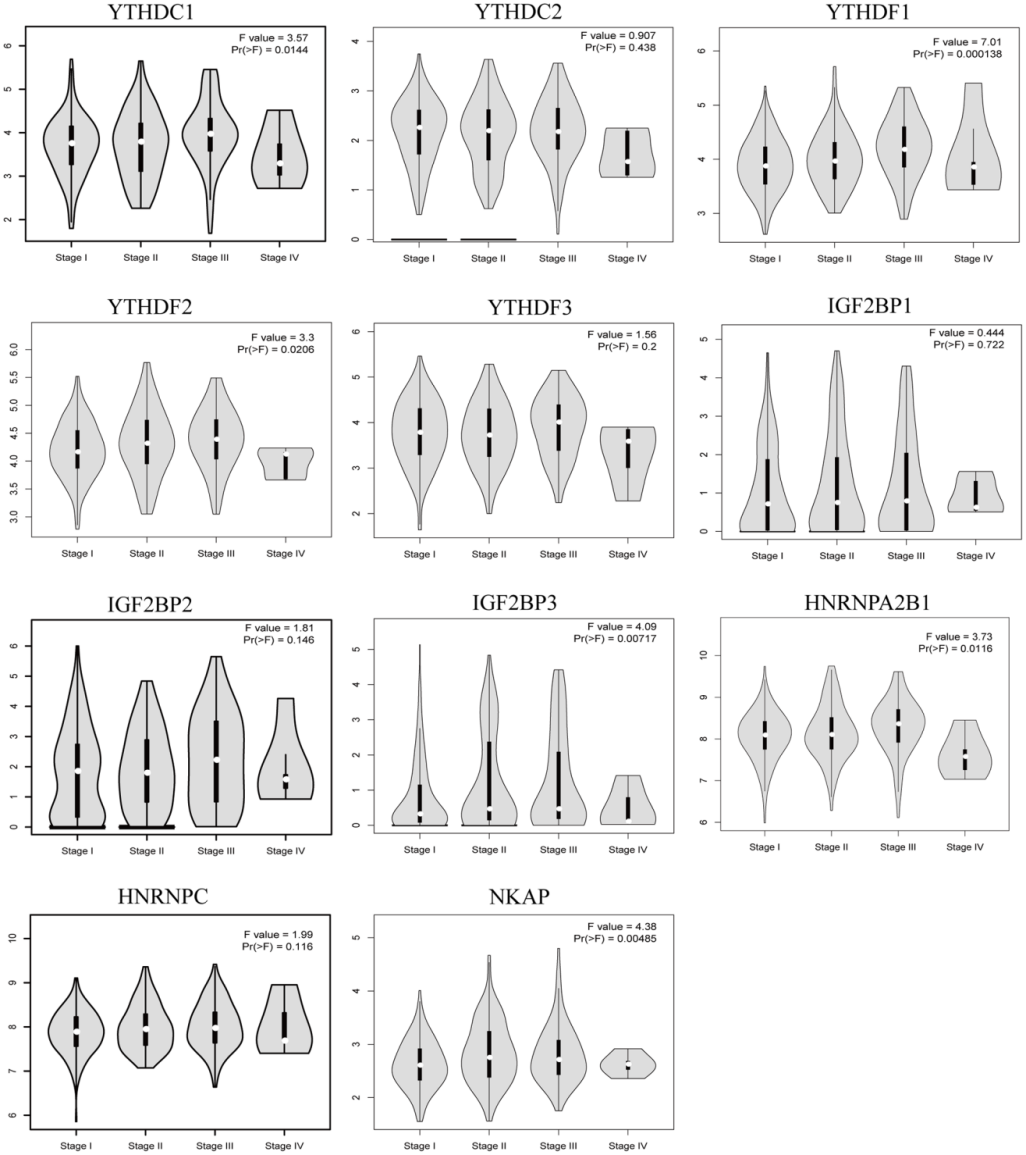
**The relationship between m6A “readers” mRNA expression and pathological stage of HCC patients (GEPIA2 database).** The mRNA expression level of YTHDC1, YTHDF1, YTHDF2, IGF2BP3, HNRNPA2B1 and NKAP were correlated with tumor stage.

**Figure 5:**
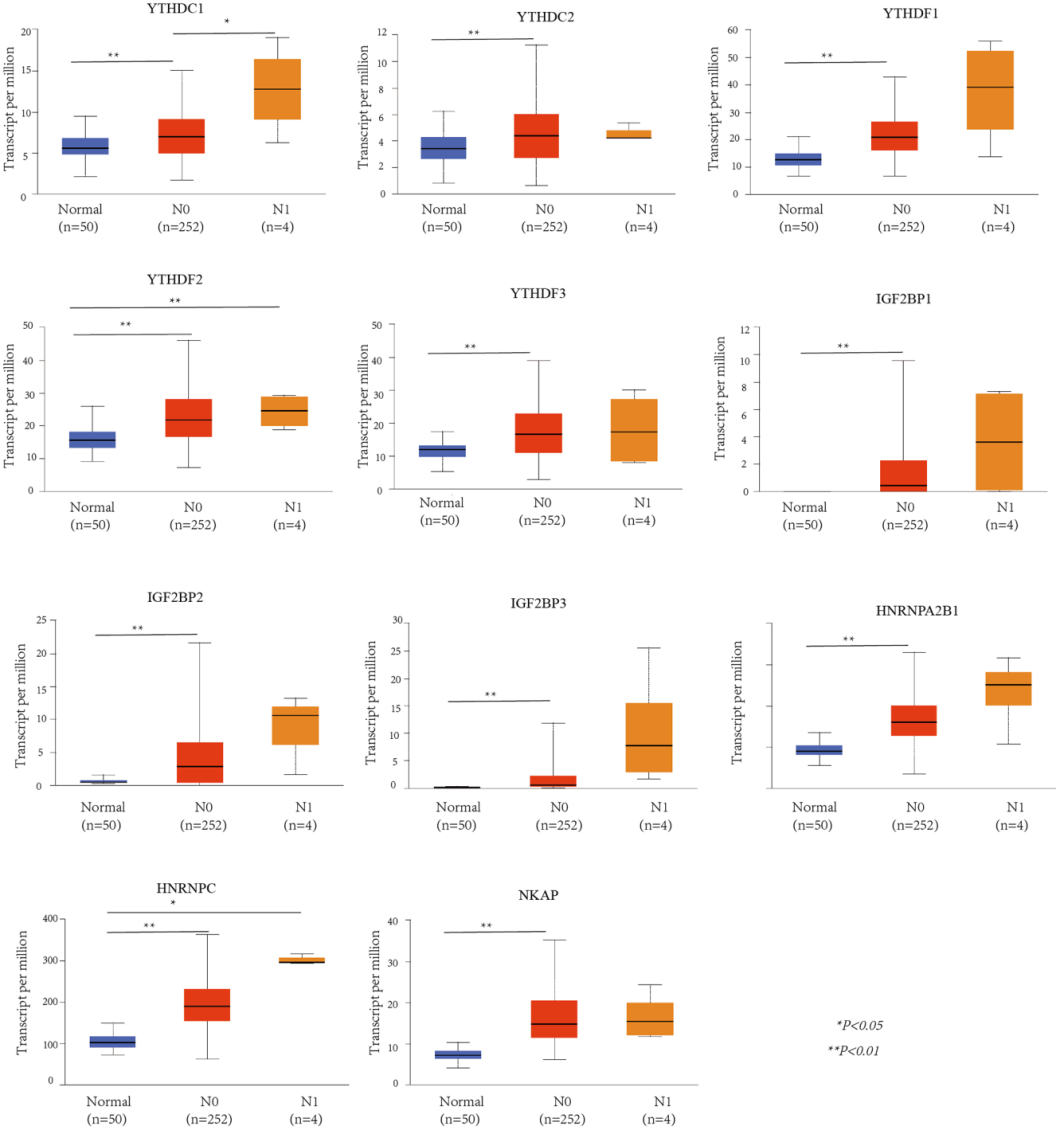
**The relationship between m6A “readers” mRNA expression and lymph node metastasis of HCC patients (UALCAN database).** YTHDF2 and HNRNPC showed significant difference between normal and N1. YTHDC1 showed significant difference between N0 and N1, while YTHDC2, YTHDF1, YTHDF3, IGF2BP1, IGF2BP2, IGF2BP3, HNRNPA2B1, and NKAP showed significant difference between normal and N0.

UALCAN was used to evaluate the association between the mRNA expression level of the m6A “readers” and the pathological stages of HCC patients (Figure 3). we used the WebGestalt database YTHDF2, YTHDF3, IGF2BP2, IGF2BP3, HNRNPA2B1 was positively correlated with tumor stage (stage1, stage2, and stage3).Additionally, the mRNA expression levels of IGF2BP1, HNRNPC and NKAP were positively correlated with tumor stage (stage1, stage2, stage3, and stage4). To further verify the correlation between m6A “readers” and tumor stage, we used GEPIA2 database to explore this correlation. And the results showed that YTHDC1, YTHDF1, YTHDF2, IGF2BP3, HNRNPA2B1, and NKAP were involved in the stage of HCC (Figure 4). Similarly, we also focused on the relationship between mRNA expression level of m6A “readers” and HCC lymph node metastasis. Propensity score matching was used to eliminate confounding factors between groups. And we found that the mRNA expression level of m6A “readers” was high in HCC patients with lymph node metastasis. But, only YTHDC1, YTHDF2 and HNRNPC showed significant correlations with HCC metastasis (Figure 5). In addition, there is a lack of data between m6A “reader” and multiple lymph node metastases in HCC, which may be due to insufficient sample size or other reasons.

### Prognostic value of m6A “readers” in HCC patients

**Figure 6:**
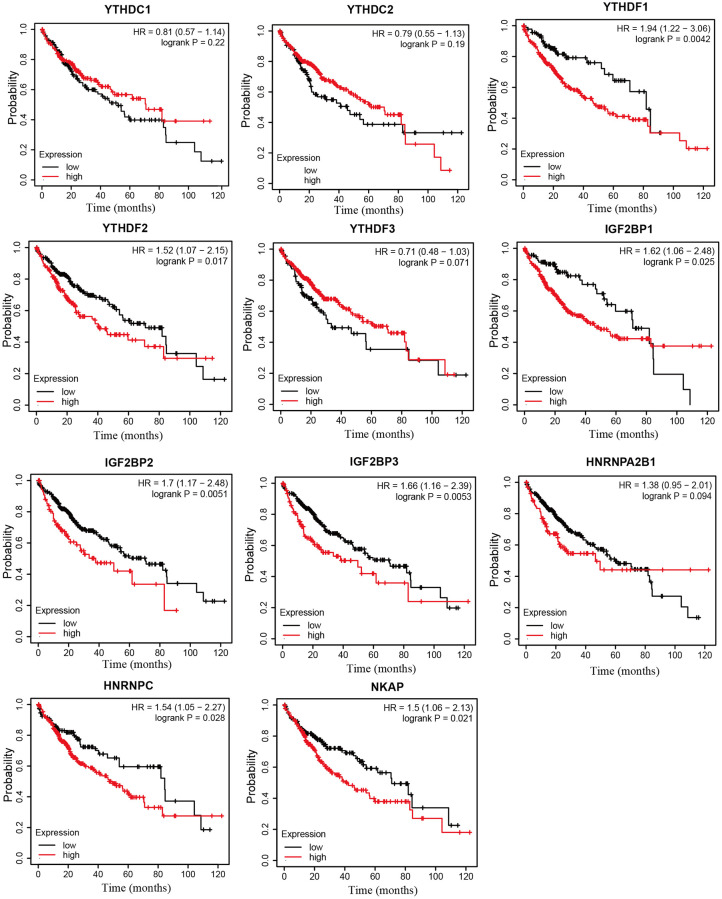
**The overall survival curve of m6A “readers” in HCC patients (Kaplan-Meier plotter database).** The high expression level of YTHDF1, YTHDF2, IGF2BP1, IGF2BP2, IGF2BP3, HNRNPC, NKAP in HCC patients displayed shorter OS time.

**Figure 7:**
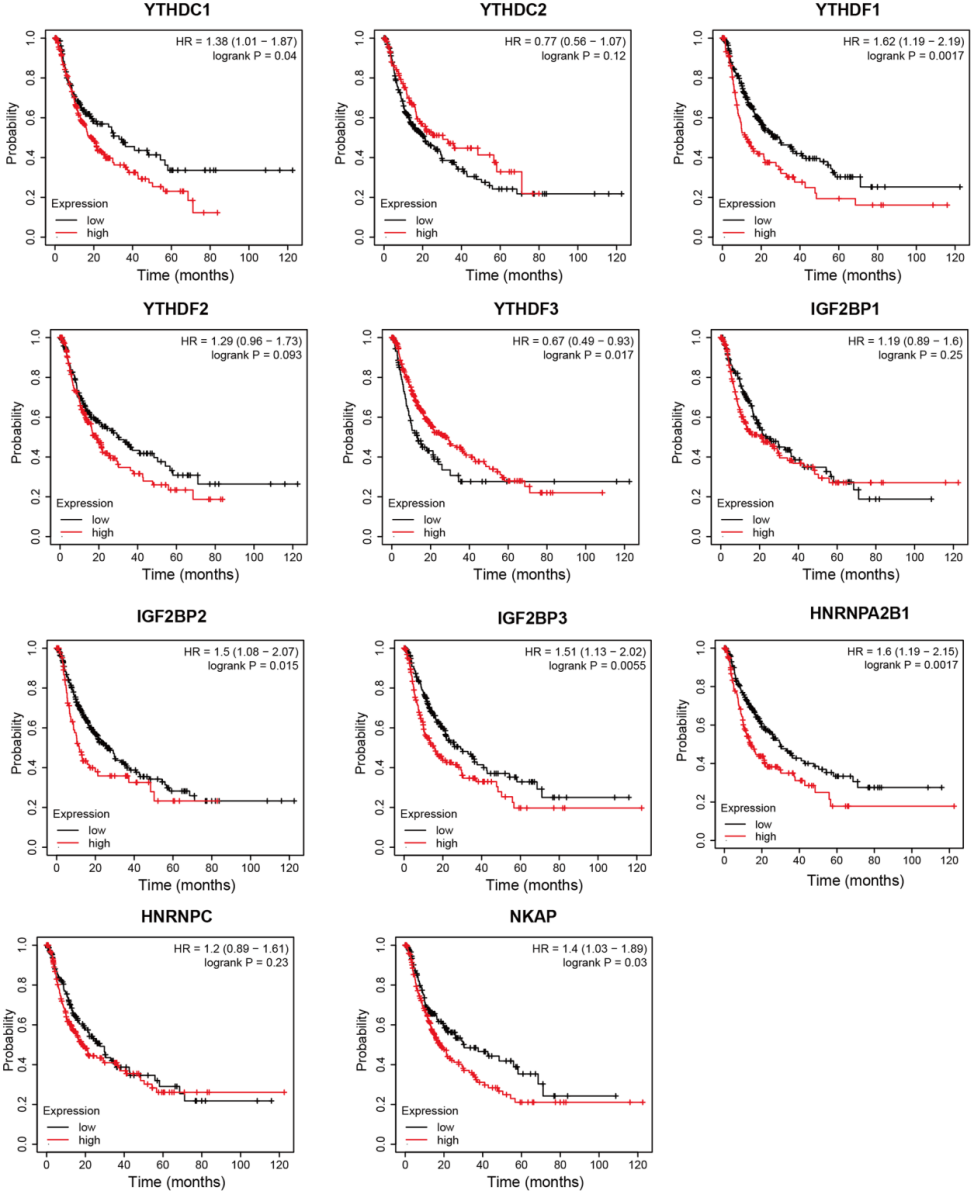
**The progression free survival curve of m6A “readers” in HCC patients (Kaplan-Meier plotter database).** YTHDC1, YTHDF1, IGF2BP2, IGF2BP3, HNRNPA2B1, and NKAP were negatively related with PFS time in HCC patients. YTHDF3 was positively related with PFS time in HCC patients.

Next, we analyzed the prognostic values of mRNA expression level of m6A “readers” in HCC patients through Kaplan-Meier plotter. It was obvious that HCC patients with higher mRNA levels of YTHDF1, YTHDF2, IGF2BP1, IGF2BP2, IGF2BP3, HNRNPC, NKAP displayed shorter overall survival (OS) time (Figure 6). The mRNA expression level of YTHDC1, YTHDF1, IGF2BP2, IGF2BP3, HNRNPA2B1, and NKAP were negatively correlated with progression-free survival (PFS) time in HCC patients. However, high expression of YTHDF3 seems to be a protective factor for PFS in HCC patients (Figure 7). Next, we combined the OS and PFS results of m6A “reader” in HCC. And we found that YTHDF1, IGF2BP2, IGF2BP3 and NKAP had significant value to predict both OS and PFS time in HCC patients.

### Genetic alteration of the m6A “readers” in HCC patients

**Figure 8:**
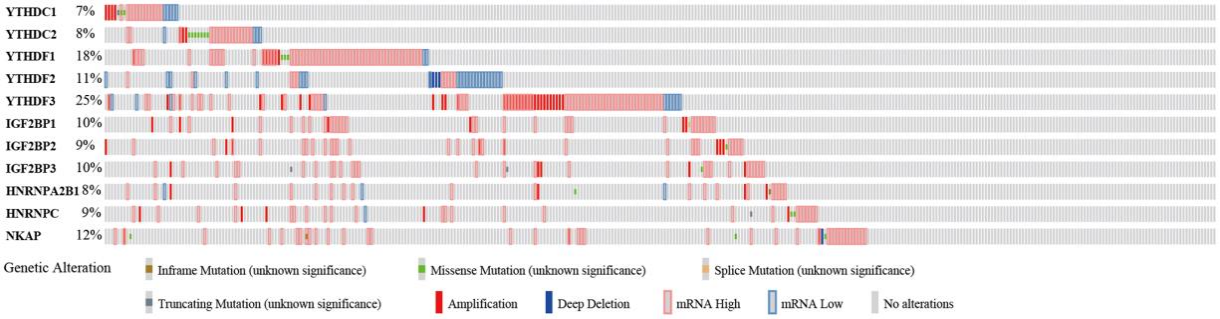
**Genetic alteration of the m6A “readers” in HCC patients (cBioPortal database).** All of these m6A “readers” had some genetic alteration, including “inframe mutation”, “missense mutation”, “splice mutation”, “truncating mutation”, “amplification”, and “deep deletion”.

To further analyze the functions of m6A “readers” in HCC patients, we focused on the alteration profiles of m6A “readers”. cBioPortal database was used to evaluate the genetic alterations of the m6A “readers” (Figure 8). According to the results in Figure 8, all of these m6A “readers” had some genetic alterations, which including “inframe mutation”, “missense mutation”, “splice mutation”, “truncating mutation”, “amplification”, “deep deletion”, “mRNA high” and “mRNA low”. And among these alterations, “mRNA high”, “mRNA low”, and “amplification” were the most common types. YTHDF3 had the highest mutation rate, with a mutation rate of up to 25%. In addition, the mutation rates of YTHDF1 (18%), YTHDF2 (11%), IGF2BP1 (10%), IGF2BP3 (10%), and NKAP (12%) were more than 10%. And the mutation rates of YTHDC1 (7%), YTHDC2 (8%), IGF2BP2 (9%), HNRNPA2B1 (8%), and HNRNPC (9%) were more than 5%.

### Interaction analyses of the m6A “readers” in HCC patients

**Figure 9:**
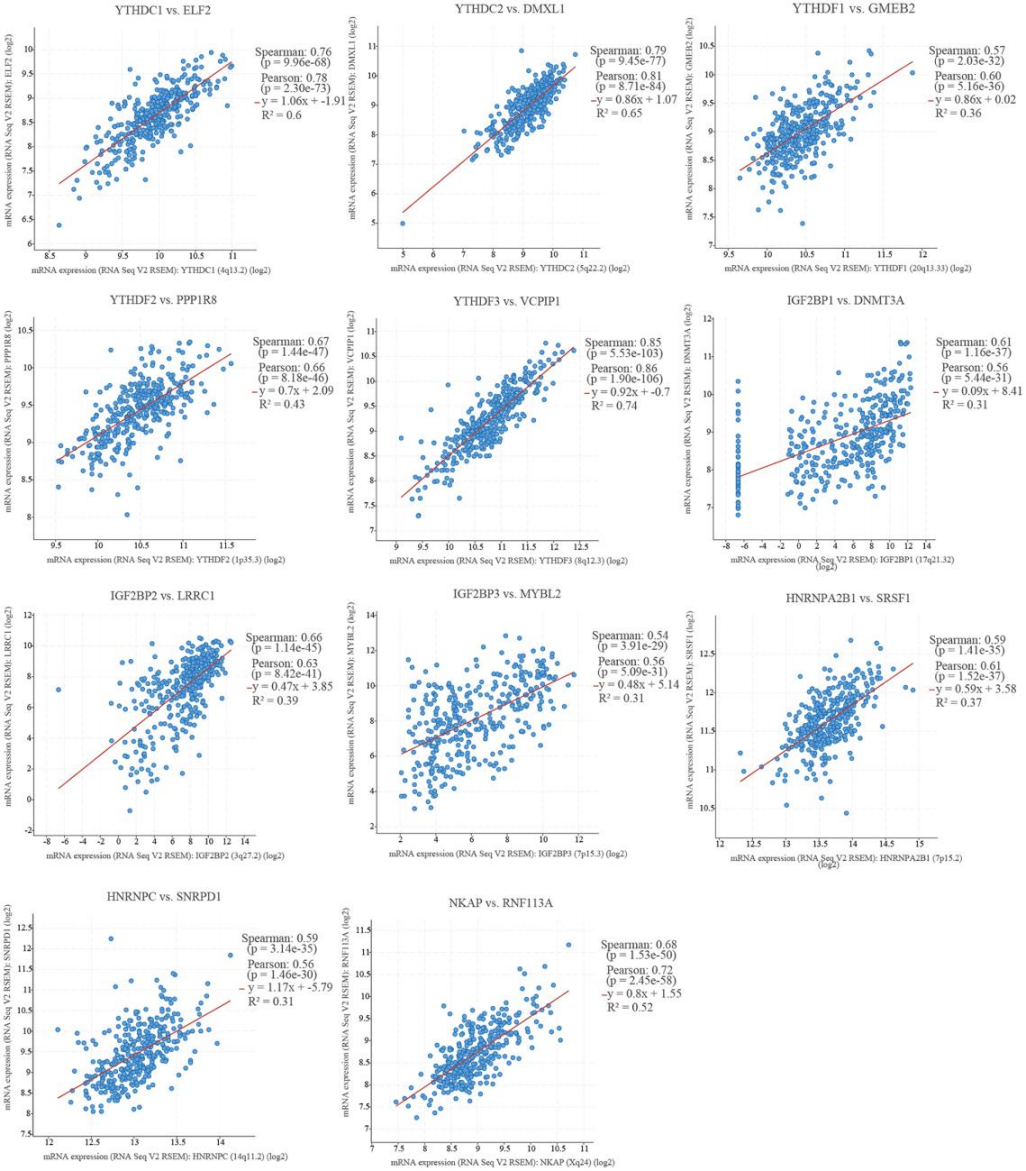
**The top one co-expression genes of m6A “readers” in HCC (cBioPortal database).** All of these m6A “readers” had a positive correlation with top one co-expression genes (including ELF2, DMXL1, GMEB2, PPP1R8, VCPIP1, DNMT3A, LRRC1, MYBL2, SRSF1, SNRPD1, and RNF113A).

**Figure 10:**
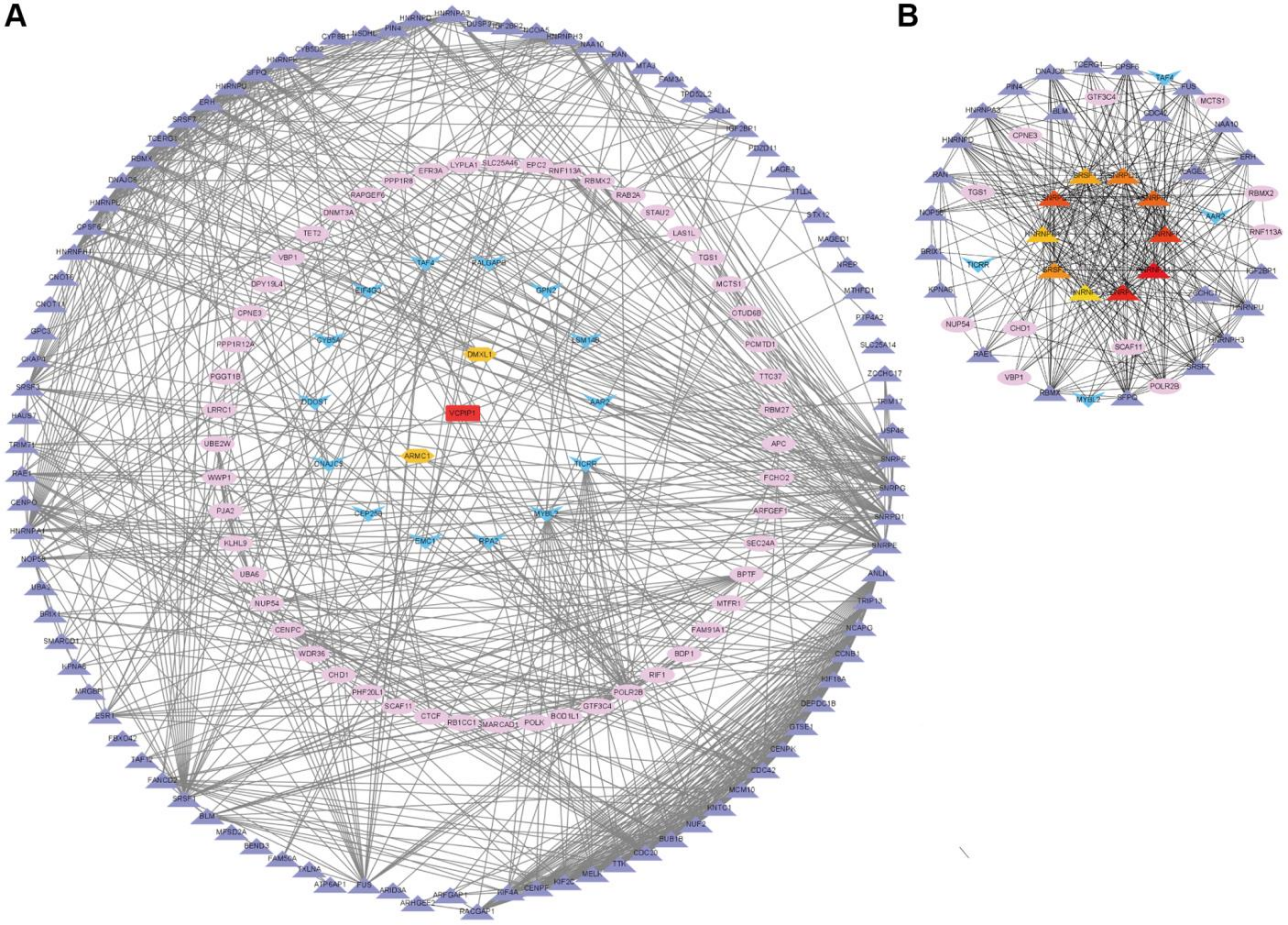
**Co-expression network analysis of the m6A “readers” related co-expression genes in HCC.** (**A**) The PPI network based on top 20 co-expression genes of each m6A “reader” from cBioPortal database. This network was edited by STRING database and Cytoscape software. The spearman's correlation value between m6A co-expression genes was depicted in different color (purple for more than 0.4, pink for more than 0.6, blue for more than 0.5, orange for more than 0.7 and red for more than 0.8). (**B**) The top 10 hub genes (little circle in the core) and their shortest paths of these co-expression genes. These data were analyzed by Cytoscape database.

To know the regulatory mechanisms of m6A “readers” in HCC, we picked out the top one co-expression genes of m6A “readers” (Figure 9). As is showed in Figure 9, ELF2, DMXL1, GMEB2, PPP1R8, VCPIP1, DNMT3A, LRRC1, MYBL2, SRSF1, SNRPD1 and RNF113A were picked out. And all of these genes present a positive correlation to m6A “readers”, with the spearman correlation coefficient greater than at least 0.4. Gene co-expression networks can help us study those genes with unknown functions [15]. So, we further explored the top 20 co-expression genes of each m6A “reader” through cBioPortal database (Supplementary Table 1). All of these co-expression genes had a high spearman’s correlation value (>0.4) with m6A “readers”. In addition, we found the spearman’s correlation value among most of these co-expression genes and m6A “readers” are more than 0.6. More importantly, the spearman’s correlation between VCPIP1 and YTHDF3 was more than 0.85. To learn the correlations among these co-expression genes, we put them into STING database. Then, we constructed a protein-protein interactions (PPIs) with these co-expression genes (Figure 10A), and found out the top 10 hub genes as well as the shortest paths (Figure 10B). As it was shown in Figure 10B, the top 10 hub genes were HNRNPA1, HNRNPK, HNRNPL, HNRNPH1, SNRPG, SNRPE, SNRPF, SNRPD1, SRSF1, SRSF3. To our surprise, these hub genes originated from three families (HNRNP family, SNRP family and SR family), which were all found to be the regulatory proteins for selective splicing.

### Functional enrichment analysis of the m6A “readers”

Functional enrichment analysis allows hundreds of genes to be allocated in different pathways thus contributing to the understanding of an unknown gene. In this study, we used the WebGestalt database to evaluate the biological functions of the m6A “readers” from pan-cancer analysis.

**Figure 11:**
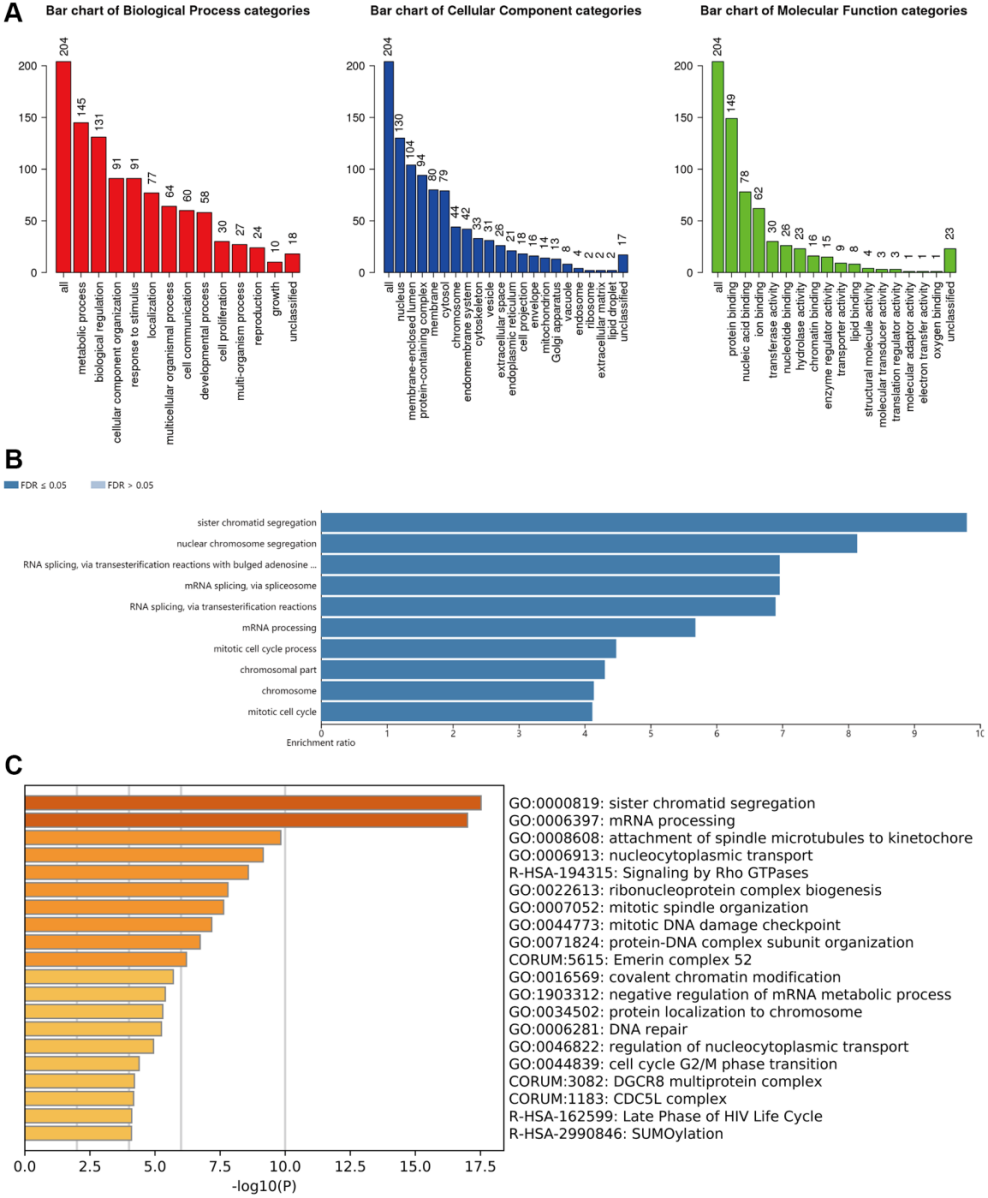
**Functional enrichment analysis of the m6A “readers” in HCC.** (**A**) GO enrichment analysis (molecular functions, biological processes and cell components) of the co-expression genes of m6A “readers”. These data were collected from WebGestalt database. (**B**) Bar graph of KEGG enrichment using WebGestalt website. The co-expressed gene of m6A “readers” were enriched in sister chromatid segregation, nuclear chromosome segregation, RNA splicing via transesterification reactions with bulged adenosine, mRNA splicing via spliceosome, RNA splicing via transesterification reactions and mRNA processing. (**C**) Bar graph of KEGG enrichment using Metascape. The co-expressed gene of m6A “readers” were enriched in sister chromatid segregation and mRNA processing pathways.

From the GO analysis, it was clearly that the most highly enriched biological process (BP) category was metabolic process, followed by biological regulation, cellular component organization and response to stimulus. In the cellular component (CC) categories, nucleus, membrane-enclosed lumen, protein-containing complex, membrane, and cytosol were highly enriched. As for the molecular function (MF) category, the co-expression genes of m6A “readers” were mainly enriched in protein binding, nucleic acid binding, and ion binding (Figure 11A).

In addition, from the KEGG pathway results (Figure 11B), it was clearly that co-expression genes were enrichment in sister chromatid segregation, nuclear chromosome segregation, RNA splicing via transesterification reactions with bulged adenosine, mRNA splicing via spliceosome, RNA splicing via transesterification reactions and mRNA processing. We found that these co-expressed genes were enriched in several splicing-related pathways. This result echoed the functions of hub genes in PPI network. Besides, we used Metascape database to confirm this result. As is shown in Figure 11C, sister chromatid segregation and mRNA processing pathways were highly enriched. The detailed information was in Table 1.

**Table 1:** Representative top 20 clusters enriched for co-expressed genes of m6A “readers”.

**GO**	**Category**	**Description**
GO:0000819	GO Biological Processes	sister chromatid segregation
GO:0006397	GO Biological Processes	mRNA processing
GO:0008608	GO Biological Processes	attachment of spindle microtubules to kinetochore
GO:0006913	GO Biological Processes	nucleocytoplasmic transport
R-HSA-194315	Reactome Gene Sets	Signaling by Rho GTPases
GO:0022613	GO Biological Processes	ribonucleoprotein complex biogenesis
GO:0007052	GO Biological Processes	mitotic spindle organization
GO:0044773	GO Biological Processes	mitotic DNA damage checkpoint
GO:0071824	GO Biological Processes	protein-DNA complex subunit organization
CORUM:5615	CORUM	Emerin complex 52
GO:0016569	GO Biological Processes	covalent chromatin modification
GO:1903312	GO Biological Processes	negative regulation of mRNA metabolic process
GO:0034502	GO Biological Processes	protein localization to chromosome
GO:0006281	GO Biological Processes	DNA repair
GO:0046822	GO Biological Processes	regulation of nucleocytoplasmic transport
GO:0044839	GO Biological Processes	cell cycle G2/M phase transition
CORUM:3082	CORUM	DGCR8 multiprotein complex
CORUM:1183	CORUM	CDC5L complex
R-HSA-162599	Reactome Gene Sets	Late Phase of HIV Life Cycle
R-HSA-2990846	Reactome Gene Sets	SUMOylation

In addition, we integrated data from Kaplan-Meier plotter database, UALCAN database and GEPIA2 database, and it was not difficult to find that YTHDF1, IGF2BP3 and NKAP were involved in OS, PFS, and stage in HCC (Supplementary Figure 1A). So, we analyzed their functional enrichment by Metascape based on the top 20 co-expression genes of them (Supplementary Figure 1B–1D). The results showed that YTHDF1 and IGF2BP3 were both related to cell cycle pathway, and NKAP was involved in protein location process. To further understand the roles of YTHDF1, IGF2BP3 and NKAP in other cancers, we collected their expression levels from pan-cancer analysis (Supplementary Figure 2A–2C). The data was obviously showed that YTHDF1, IGF2BP3 and NKAP were highly expressed in most cancer types.

### Immune cell infiltration of the m6A “readers” in HCC

**Figure 12:**
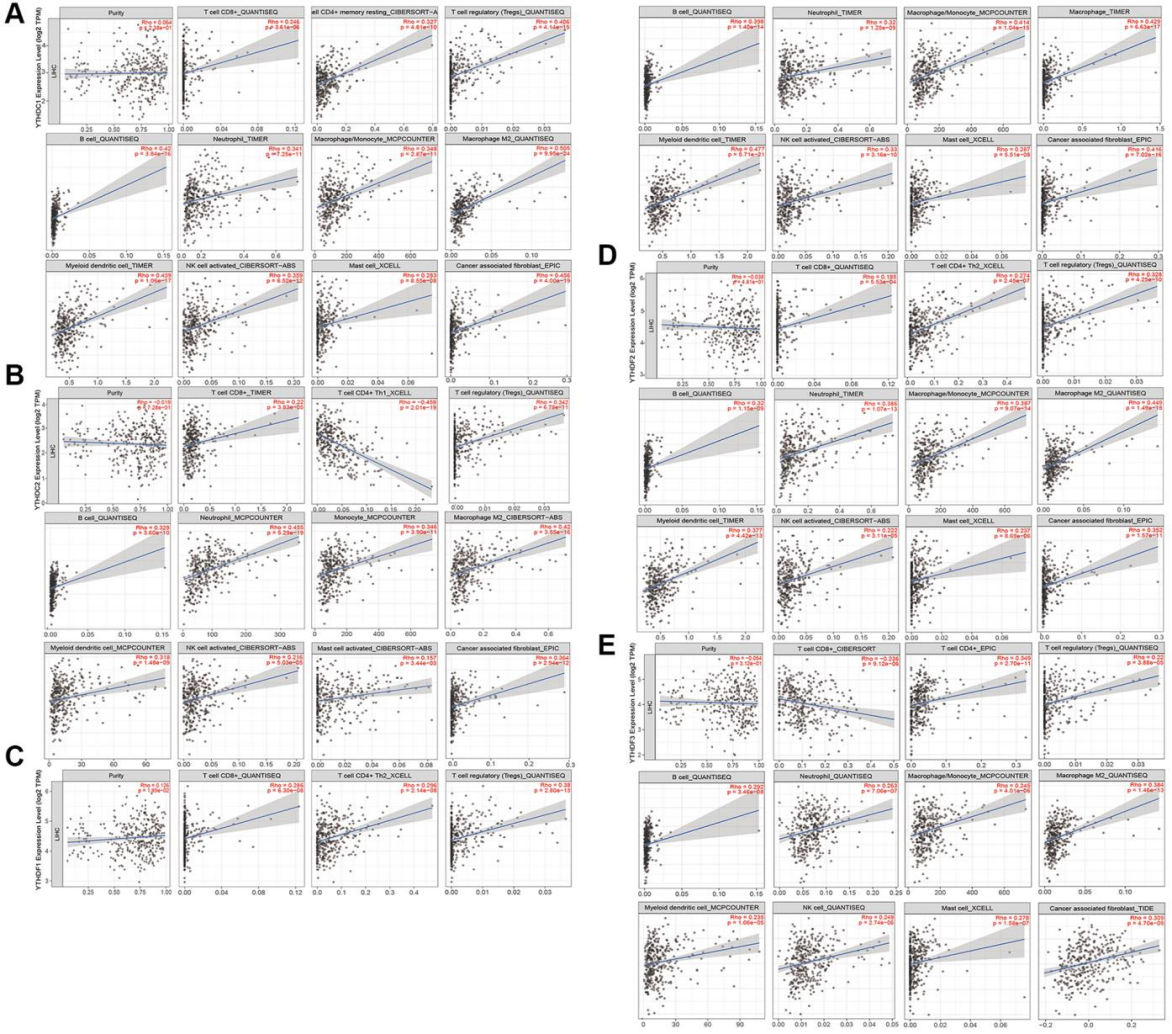
**Immune cell infiltration of the m6A “readers” (YTHDC1/2, and YTHDF1/2/3) in HCC.** (**A**) All of these 11 immune cells had a positive correlation with YTHDC1. (**B**) All these immune cells we researched had positive correlations with YTHDC2, except for CD4+ T cell. (The CD4+ T cell was negatively related to YTHDC2). (**C**) All of these 11 immune cells had a positive correlation with YTHDF1. (**D**) All of these 11 immune cells had a positive correlation with YTHDF2. (**E**) All these immune cells we researched had positive correlations with YTHDF3, except for CD8+ T cell. (The CD8+ T cell was negatively related to YTHDF3). These data were collected from TIMER 2.0 database.

To explore the correlation between the m6A “readers” and immune cell infiltration in HCC, we searched some information from the TIMER 2.0 database (Figure 12 and Supplementary Figures 3, 4). As is shown, we discussed 11 types of immune cells including CD8+ T cell, CD4+ T cell, Tregs, B cell, neutrophil, monocyte, macrophage, myeloid DC, NK cell, mast cell, and CAF. Tumor purity is a major confounding factor in immune cell infiltration analysis. Therefore, all of our results had a purity adjustment. And in general, most of these 11 immune cells had a positive correlation with m6A “readers”, such as YTHDC1 (Figure 12A), YTHDF1 (Figure 12C), YTHDF2 (Figure 12D), IGF2BP1 (Supplementary Figure 3A), IGF2BP2 (Supplementary Figure 3B), HNRNPA2B1 (Supplementary Figure 4A), HNRNPC (Supplementary Figure 4B) and NKAP (Supplementary Figure 4C). However, the CD4+ T cell was negatively related to YTHDC2 (Figure 12B); the CD8+ T cell was negatively related to YTHDF3 (Figure 12E); the mast cell was negatively related to IGF2BP3 (Supplementary Figure 3C). In addition, there were no significant difference between IGF2BP1 and neutrophil. When we explored the relationship between immune cells and m6A “readers” such as IGF2BP3 and NKAP, the TIMER 2.0 database mixed macrophages and monocytes together for analysis. These may because some subpopulations of macrophages originated from adult monocytes [16]. In addition, we found that macrophages had a positively strong correlation (Rho>0.4) with most m6A “readers”, such as YTHDC1, YTHDC2, YTHDF1, YTHDF2, IGF2BP3, HNRNPA2B1, and NKAP. Besides, CD4+ T cell had a positively strong correlation with YTHDC2, IGF2BP2, IGF2BP3, and HNRNPC; Treg had a positively strong correlation with YTHDC1, IGF2BP2, HNRNPA2B1, and HNRNPC; B cell had a positively strong correlation with YTHDC1, HNRNPA2B1, and HNRNPC, monocyte had a positively strong correlation with YTHDF1, IGF2BP3, HNRNPA2B1, and HNRNPC, and DC had a positively strong correlation with YTHDC1, YTHDF1, IGF2BP3, HNRNPA2B1, and HNRNPC; Neutrophil had a positively strong correlation with YTHDC2; CAF had a positively strong correlation with YTHDC1 and YTHDF1; while CD8+ T cell, NK, and mast cell existed a much weaker correlation with all of these m6A “readers”. HNRNPC, HNRNPA2B1 and YTHDC1 had positive correlation with more than four immune cells, including Tregs, B cell, macrophage, DC and so on. However, YTHDF3, IGF2BP1, and NKAP showed weaker correlation with all of the immune cells we researched. More importantly, we obtained the most related immune cells of m6A “readers”. For example, the most related immune cell of HNRNPC was myeloid dendritic cell (Rho = 0.535). More detailed information was shown in Table 2 (the correlation coefficients greater than 0.4 have been bolded, and the negative correlation coefficients have been bolded and italicized).

**Table 2:** The spearman's correlation value between the m6A “readers” and immune cells.

	**T cell CD8+**	**T cell CD4+**	**Tregs**	**B cell**	**Neutrophil**	**Monocyte**	**Macrophage**	**DC**	**NK cell**	**Mast cell**	**CAF**
YTHDC1	0.246	0.327	**0.406**	**0.420**	0.341	0.348	**0.505**	**0.439**	0.359	0.283	**0.456**
YTHDC2	0.220	**0.459**	0.342	0.329	**0.455**	0.346	**0.420**	0.318	0.216	0.157	0.364
YTHDF1	0.286	0.296	0.380	0.398	0.320	**0.414**	**0.429**	**0.477**	0.330	0.287	**0.416**
YTHDF2	0.185	0.274	0.328	0.320	0.386	0.387	**0.449**	0.377	0.222	0.237	0.352
YTHDF3	***−0.236***	0.349	0.220	0.292	0.263	0.245	0.384	0.235	0.249	0.278	0.309
IGF2BP1	0.172	0.263	0.264	0.334	**−**	0.255	0.272	0.280	0.225	0.252	0.142
IGF2BP2	0.255	**0.418**	**0.408**	0.381	0.127	0.346	0.346	0.348	0.237	0.223	0.269
IGF2BP3	0.273	**0.464**	0.375	0.304	0.110	**0.416**	**0.416**	**0.401**	0.281	***−0.165***	**−0.236**
HNRNPA2B1	0.297	0.381	**0.412**	**0.439**	0.319	**0.440**	**0.464**	**0.476**	0.391	0.312	0.374
HNRNPC	0.340	**0.453**	**0.414**	**0.473**	0.316	**0.400**	**0.417**	**0.535**	0.367	0.370	0.382
NKAP	0.227	0.316	0.219	0.311	0.231	0.355	0.355	0.372	0.298	0.143	0.263

### Validation the expression level and functions of IGF2BP3 in HCC cell lines

**Figure 13:**
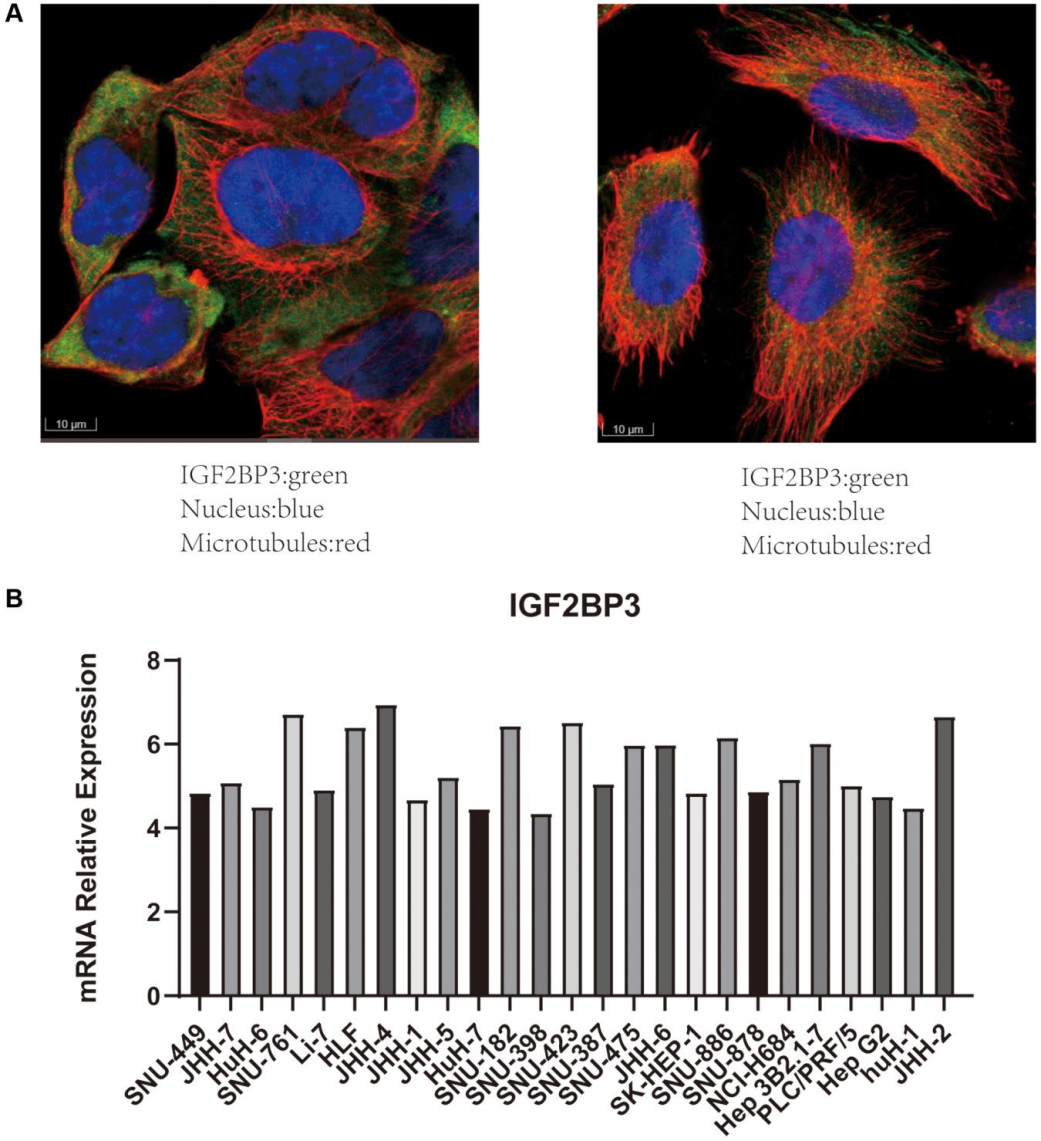
**Validation the expression of IGF2BP3 in cell line models.** (**A**) The immunofluorescence results of IGF2BP3. IGF2BP3 was localized in the cytoplasm. (IGF2BP3 was stained in green, the nucleus was stained in blue, and the microtubules were stained in red.) The data were derived from the HPA database. (**B**) The mRNA expression level of IGF2BP3 in HCC cell lines. The data were derived from CCLE database.

To verify the reliability of the results, we carried out further studies using IGF2BP3 as a representative gene (because of its association with OS, PFS, and stage in HCC). First, we used immunofluorescence results from HPA database and demonstrated that IGF2BP3 was mainly localized in the cytoplasm (Figure 13A). IGF2BP3 was stained in green, the nucleus was stained in blue, and the microtubules were stained in red. This immunofluorescence information was based on A-431 (Figure 13A left) and U-251 (Figure 13A right) cell lines. In addition, CCLE database was used to display the mRNA expression level of IGF2BP3 in multiple HCC cell lines (Figure 13B).

**Figure 14:**
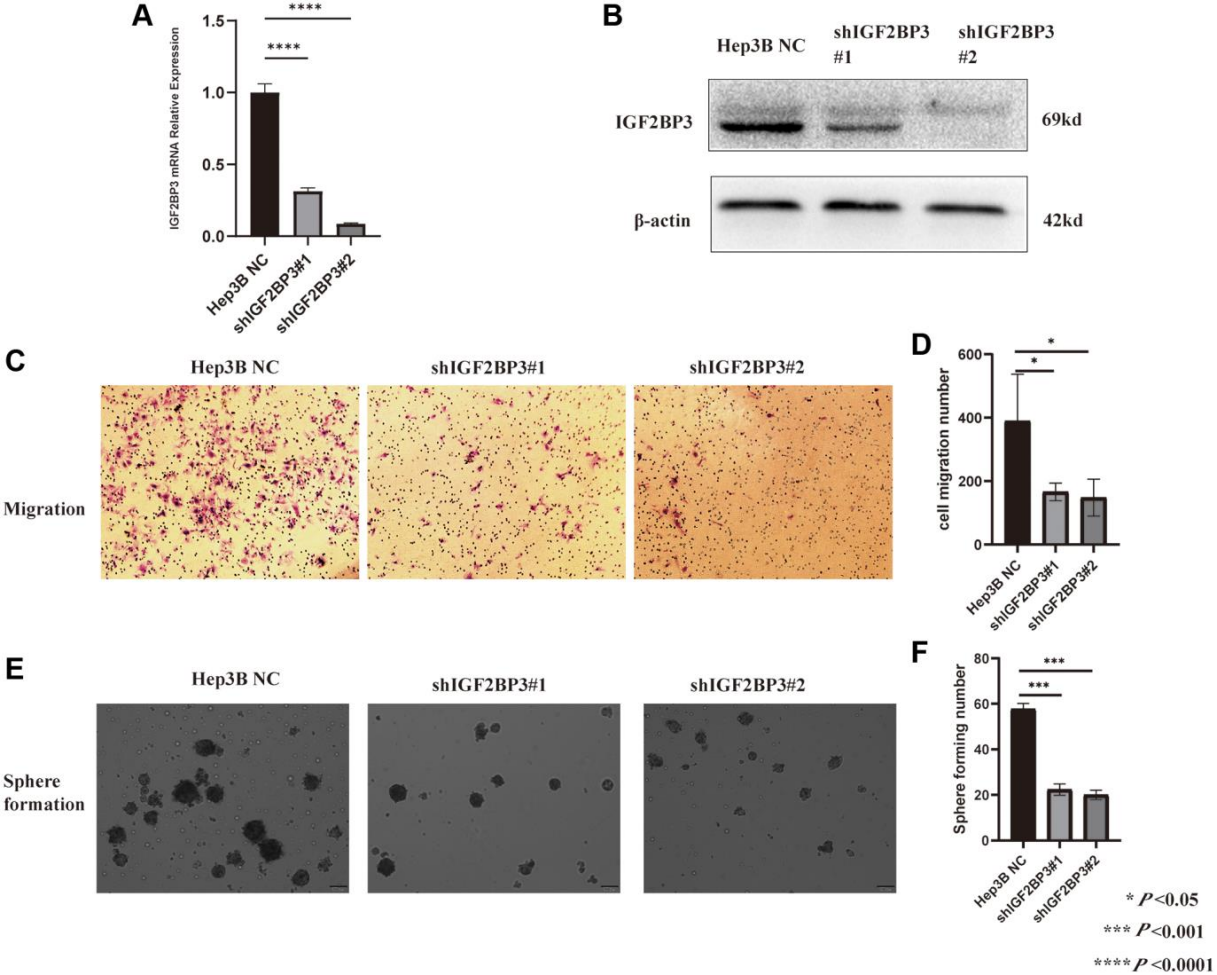
**Validation the functions of IGF2BP3 in HCC cell line.** (**A**) The knock-down efficiency of IGF2BP3 mRNA expression in Hep3B cell. (**B**) The knock-down efficiency of IGF2BP3 protein expression in Hep3B cell. (**C**) The representative migration images of IGF2BP3 stably KD and NC Hep3B cells. (**D**) The statistical analysis of migration assay for Hep3B cells with IGF2BP3 stably KD and NC. ^*^P < 0.05; ^***^P < 0.001; and ^****^P < 0.0001. (**E**) The representative sphere formation assay images of IGF2BP3 stably KD and NC Hep3B cells. (**F**) The statistical analysis of sphere formation assay for Hep3B cells with IGF2BP3 stably KD and NC. ^*^P < 0.05; ^***^P < 0.001; and ^****^P < 0.0001.

Based on the expression level of IGF2BP3 from CCLE database, we stably knocked down the IGF2BP3 mRNA level in Hep3B cell, and the knocked-down (KD) efficiency were checked by RT-PCR (Figure 14A) and Western Blot (Figure 14B). In addition, the migration rate of IGF2BP3 stably KD cells were significantly reduced compared to the non-targeted control (NC) (Figure 14C, 14D). Besides, we performed the sphere formation assay to verify stemness characteristics of IGF2BP3 in HCC. As it was showed in Figure 14E, 14F, IGF2BP3 stably KD cells showed significantly reduced sphere formation capacity.

## DISCUSSION

The biological importance of m6A modifications mainly depends on the roles of m6A “readers” which control RNA fate and function. Studies showed that some m6A “readers” were involved in the development of HCC. For instance, Tanabe A and his colleague proved that through activation of c-Jun and ATF-2, YTHDC2 played a significant role in promoting HCC cell growth [17]. In addition, Liu et al. implicated that YTHDC2 might be a target to improve the therapeutic efficacy for HCC patients [14]. Zhao et al. believed that YTHDF1 could serve as independent prognostic factors for HCC through bioinformation analysis [18]. Liu et al. revealed that YTHDF1 accelerated the translational output of FZD5 mRNA and promoted the progression of HCC through the WNT/β-catenin pathway [19]. Zhong et al. found that YTHDF2 could be a tumor suppressor to inhibit cell proliferation and growth in HCC through promoting the degradation of EGFR mRNA [20]. In addition, YTHDF2 has also been reported to promote stem cell phenotype and metastasis of HCC through regulating the m6A methylation level of OCT4 mRNA [21]. Pu et al. indicated that IGF2BP2 could maintain FEN1 expression in an m6A-IGF2BP2-dependent method, and it may be a potential biomarker for the prognostic prediction in HCC [22]. However, no comprehensive analysis based on multiple m6A “readers” in HCC has been integrated so far.

In this study, we conducted a comprehensive study on the expression level and clinical prognostic value of m6A “readers” in HCC. We showed the different expression of m6A “readers” between HCC and normal tissues in mRNA and protein level. Our results showed that the mRNA expression level of the common m6A “readers” including YTHDC1, YTHDC2, YTHDF1, YTHDF2, YTHDF3, IGF2BP1, IGF2BP2, IGF2BP3, HNRNPA2B1, HNRNPC, and NKAP were highly expressed in HCC. And the protein expression level of most of m6A “readers” was the same as mRNA expression. In addition, YTHDC1, YTHDF1, YTHDF2, IGF2BP3, HNRNPA2B1 and NKAP were confirmed by two databases to be positively correlated with tumor stage, which deserved more attention in the treatment of HCC. Besides, our results implied that YTHDC1, YTHDF1, YTHDF2, IGF2BP1, IGF2BP2, IGF2BP3, HNRNPA2B1, HNRNPC, and NKAP had a prognosis value in either OS or PFS. But only YTHDF1, IGF2BP2, IGF2BP3 and NKAP had significant value to predict both OS and PFS time in HCC patients. This seems a little inconsistent with the previous results [14]. Due to the researches on YTHDC2 in HCC were really rare, we could not interpret this paradoxical result. More mechanism and clinical researches were needed.

Somatic mutation and clonal selection were two major reasons for cancer development [23, 24]. There were large amounts of evidences showed that genetic alterations play a significant role in tumor occurrence [25, 26]. Researches showed that there are two categories of clinically important gene mutations. The first category is single nucleotide mutations (including synonymous mutations, missense mutations, nonsense mutations and splice mutation), which involves mutations that alter the coding region of a gene, resulting in the loss of function or abnormal activation of the protein product of a gene. The second type of mutation is structural mutations (including deletions, insertions, duplications, inframe mutation, amplifications, and inversions), which may involve the non-coding sequences that make up 98% of the genome [27]. In this study, all of the m6A “readers” shows frequent gene alterations in HCC. The major genetic alteration is the change of mRNA expression level. In addition, some clinically important gene mutations such as nonsense mutations, splice mutation, inframe mutation, amplifications, and deletions, are also accounted for a certain percentage. These results partly explained the genetic alteration of the m6A “readers” probably played a critical role in HCC.

The co-expression genes may give some new thoughts to the development and progression of HCC. For instance, they may be potential targets of m6A “readers”, or be regulators of m6A “readers”. To determine the functions of these m6A “readers” in HCC, we depicted a PPI network based on the co-expression genes of m6A “readers”. The top 10 hub genes seem from three families (HNRNP family, SNRP family, and SR family). In addition, HNRNP, SNRP, and SR proteins were all discovered as regulators of alternative splicing. HNRNP family was a large family of RNA-binding proteins (RBPs) that participated in multiple aspects of nucleic acid metabolism, which including alternative splicing, mRNA stabilization, transcriptional regulation and translational regulation [28]. Small nuclear ribonucleoprotein polypeptide (SNRP) family is precursors of spliceosome [29, 30]. And they have attracted large attention because of their implicated roles in tumorigenesis and tumor development [31–33]. Serine and arginine-rich (SR) family were known as constitutive and alternative splicing regulators. Shuttling SR family had a potential to be regulators for translation in the cytoplasm [34]. Besides, SR and HNRNP family were discovered as alternative splicing regulators. SR and HNRNP family significantly affect the responses to chemotherapy through acting as modulators of drug-induced apoptosis [35]. In conclusion, the function of m6A “readers” on HCC may link with these splicing related proteins, and these families may have a potential to be biomarkers or therapeutic targets.

The GO and KEGG enrichment analysis helped us to understand the functional meanings of genes at a molecular level [36, 37]. And according to our enrichment analysis, the metabolic process and RNA splicing associated pathway were enriched. These results, to a certain extent, reconfirmed the critical role of splicing related mechanisms of m6A “readers” in HCC.

In recent years, immunotherapy becomes a popular research field. It is noteworthy that clinically detected cancers must evade the anti-tumor immune response before they can grow gradually. Tumor immune monitoring is essential for the body to eliminate tumor cells. Studies have shown that tumors can resist immune attack through immune system suppression pathways and immune system exclusion or neglect [38]. The tumor progression and recurrence may be associated with immune cell infiltration, and comprehending the interaction between the tumor and host immune system is essential for the discovery of prognostic biomarkers, the reduction of drug resistance and the development of new therapeutic approaches [39]. Furthermore, in previous researches, many immune cells played a key role in the development of tumors. For instance, dendritic cell immunotherapy has been shown to be effective against tumors beyond the central nervous system [40]. CD8+ T cell can destroy tumor cells by cytotoxic molecules such as granzyme and perforin [41]. The goal of cancer immunotherapy is to promote the activity of cytotoxic T lymphocytes (CTL) within the tumor and to establish effective and durable anti-tumor immunity. Specific dendritic cells will help signal transmission from T cells CD4+ to T cells CD8+ to optimize the size and quality of the CTL response [42, 43]. Furthermore, studies have shown that B cells and B cell receptor-associated kinases, such as BTK, function in the microenvironment of squamous cell carcinoma and pancreatic cancer, so targeting B cells or B cell receptor-associated kinases may have more potent anticancer activity than B cell malignancies [44]. In addition, m6A methylation also was reported to modulate immunity and predict patient outcomes [45, 46]. Our study exhibited the relationship between m6A “readers” and immune cell infiltration in HCC.

We found that the expression of the m6A “readers” is significantly positively linked to the infiltration of CD8+ T cell, CD4+ T cell, Tregs, B cell, neutrophil, monocyte, macrophage, DC, NK cell, and mast cell. It was worthy to pay attention to the correlation between HNRNPC and DC. They had a high correlation value 0.535. In addition, among these immune cells, macrophage might play a core role in m6A “readers”, because it showed positively strong correlation with most m6A “readers”. Macrophage was reported as potent immune effector cells, and it had a functional plasticity which contributed to both antitumor and protumor function in different background [47]. In this study, macrophage acted as a protumor factor and the detailed mechanisms were worthy of digging. Besides, the distinct changes in the microenvironment may induce a series of reversible metabolic programs which might form the basis of the activation of macrophage [48]. Thus, m6A “readers” may exert their functions in the microenvironment by interacting with macrophages. However, it needs more researches to clarify the mechanisms. In addition, m6A “readers” also interact with various immune cells. For instance, more than six immune cells showed positively strong correlation with HNRNPC. These results suggested that m6A “readers” played a key role in promoting tumor progression through modulating tumor immune microenvironment [49].

In further analysis and validation of IGF2BP3 functions in cell model, we found that IGF2BP3 showed high expression in HCC cell lines according to the CCLE database, and knockdown of IGF2BP3 inhibited the migration and sphere formation ability of HCC cells. These results suggested that IGF2BP3 might act as an oncogene and was related to the poor prognosis of HCC patients by enhancing cell migration and stemness ability.

### Conclusions and limitations

In this study, we systematically collated the expression data of the common m6A “readers” in HCC, and analyzed their clinical value. In addition, we speculated their possible functions and discussed their correlation with immune cell infiltration. It was worthy to note that YTHDF1, IGF2BP3 and NKAP may be potential therapeutic targets and biomarkers. Because they were expressed differently, and were negatively correlated to OS, PFS, and tumor stage in HCC. More importantly, YTHDF1, IGF2BP3 and NKAP were also highly expressed in pan-cancer, which provided the promise of YTHDF1, IGF2BP3 and NKAP as potential therapeutic targets. From the results of the functional enrichment analysis, the main functions of these three potential therapeutic targets are involved in the regulation of cell cycle or protein localization. In addition, the functions of m6A “readers” in HCC may be related to splicing proteins such as HNRNP, SNRP and SR. And the immune cells especially the macrophage, CD4+ T cell, Treg, B cell, monocyte, and myeloid dendritic cell may play a significant role in the progression of HCC. Besides, immune microenvironment of m6A “readers” was also worthy to be explored. In conclusion, our findings in this study could give some new idea to the development of multiple molecular diagnostic biomarkers and treatment targets for HCC patients to improve their prognosis.

Protein expression patterns in cancers contain crucial diagnostic and prognostic information. However, from the HPA database, we could not obtain the protein expression information of YTHDF1 and YTHDF3 in HCC. In addition, there was no data to present the correlations between m6A “readers” and metastasis of multiple lymph node in HCC, and it may be caused by insufficient sample size or other reasons. Besides, we found that most of the m6A readers were associated with the prognosis of HCC patients, and the m6A “readers” were found to be closely related to the immune microenvironment. So, in the near future, we will compose a prognostic model incorporating the m6A “readers” (especially YTHDF1, IGF2BP3 and NKAP) and immune microenvironment components (especially the macrophage, CD4+ T cell, Treg, B cell, monocyte, and myeloid dendritic cell) to better complement this study. Furthermore, our research was mainly based on public databases, and the results of this study should be further validated by more laboratory experiments.

## MATERIALS AND METHODS

Multiple bioinformatics database resources were accessed to explore the expression levels of m6A “readers” in HCC samples. The website tools (UALCAN, GEPIA2, and cBioPortal databases) were based on the TCGA database, which contains 371 HCC samples and 50 normal liver samples. And TPM (Transcripts per million) data was applied for the mRNA expression level analysis.

### UALCAN database

The UALCAN database is an interactive portal for in-depth analysis of gene expression data from the TCGA database. It compares the tumor and normal tissue samples based on pathological stage, lymph nodes metastasis and other clinicopathological characteristics [50]. UALCAN database was available from http://ualcan.path.uab.edu/index.html. We used UALCAN database to evaluate the expression difference between HCC and normal tissue. And the differences in transcriptional expression were compared by Student’s *t*-test, we considered *P* < 0.05 as statistically significant. In addition, the figures of pathological stage and lymph nodes metastasis were also derived from this database.

### The human protein atlas (HPA) database

The Human Protein Atlas database is an immunohistochemistry-based map of protein expression profiles in normal tissues, cancers and cell lines. Proteome analysis was based on 26941 antibodies targeting 17165 unique proteins. It allows complex queries (including expression profiles, protein classes and chromosome location), so as to provide a resource for pathology-based research [51]. And the Human Protein Atlas was available from http://www.proteinatlas.org. In this study, we used the HPA database to show the different expression of m6A “readers” between HCC and normal tissues.

### GEPIA2 database

GEPIA2 was available to analyze gene expression quantification both in the gene level and the transcript level. In addition, it was able to analyze and compare different cancer subtype [52]. GEPIA2 analyzed RNA sequencing data from the GTEx and TCGA database, and it was publicly accessible at http://gepia2.cancer-pku.cn/. In this study, we used GEPIA2 database to show the correlation between m6A “readers” and HCC stage.

### Kaplan-Meier plotter

The Kaplan Meier plotter could evaluate the effect of 54k genes (mRNA, miRNA, protein) on survival in 21 cancer types. The correlations between gene expression and survival were computed using the Cox proportional hazards regression. And the sources for the databases contain GEO, EGA, and TCGA [53]. Kaplan-Meier plotter was available from http://kmplot.com/analysis/. We used Kaplan-Meier plotter to analyze the prognostic value including overall survival (OS) and progression free survival (PFS) of the m6A “readers” in HCC patients. We divided 371 HCC specimens into high and low expression groups according to their mRNA expression by automatically selecting the best cut-off value. Kaplan-Meier analysis was evaluated by log-rank test, and we considered *P* < 0.05 as statistically significant difference.

### cBioPortal

cBioPortal database provides a Web resource for exploring, visualizing, and analyzing cancer genomics data. It provides and simplifies the summaries of gene-level data from multiple platforms [54]. cBioPortal was available from https://www.cbioportal.org. In this study, we used cBioPortal to analyze the genome map of the m6A “readers” in HCC and obtain the mutation and mRNA expression data. Besides, we downloaded the co-expression genes of m6A “readers” from cBioPortal for further analysis.

### STRING

The STRING database collects and integrates publicly available sources of protein-protein interaction information, and provides the computational predictions. And it aims to achieve a comprehensive and objective global network among proteins [55]. STRING was available from https://string-db.org/. In our study, STRING was applied to explore and analyze possible protein-protein interactions (PPIs).

### Cytoscape

Cytoscape is a popular tool for analyzing and visualizing multiple proteins or genes and for integrating biomolecular interaction networks into a unified conceptual framework [56]. In our study, we used Cytoscape to conduct functional integration on 220 co-expression genes of m6A “readers” screened from cBioPortal. These 220 co-expression genes integrated from top 20 genes that had a high spearman’s correlation with m6A “readers”. According to the spearman’s correlation value between the interacting proteins, we made genes with high spearman’s correlation value (>0.6) pink, and those genes with low spearman’s correlation value (<0.6) blue. Besides, we used it to figure out top 10 hub genes of these co-expression genes.

### WebGestalt

WebGestalt is a tool for the interpretation of gene lists. It supports three well-established and complementary methods for enrichment analysis, including over-representation analysis (ORA), gene set enrichment analysis (GSEA), and network topology-based analysis (NTA) [57]. WebGestalt was available from http://www.webgestalt.org/. In our study, we used WebGestalt to provide enrichment analysis results including Gene ontology (GO) enrichment analysis and Kyoto Encyclopedia of Genes and Genomes (KEGG) pathway analysis of co-expression genes of m6A “readers”.

### Metascape

Metascape, a gene annotation and analysis resource, integrated functional enrichment, interactome analysis, gene annotation, and membership together [58]. Metascape was available from https://metascape.org/gp. In this study, we used Metascape to analyze the functional enrichment of m6A “readers” and their co-expression genes.

### TIMER2.0

TIMER is a comprehensive resource for the analysis of immune infiltrates among various cancer types. TIMER2.0 provides immune infiltrates’ abundances estimated by multiple immune deconvolution methods [59]. TIMER2.0 was available from http://timer.cistrome.org/. In this study, we used TIMER2.0 to depict the relationship between m6A “readers” and immune cells including CD8+ T cell, CD4+ T cell, T cell regulatory (Tregs), B cell, neutrophil, monocyte, macrophage, myeloid dendritic cell (DC), nature killer (NK) cell, mast cell, and cancer associated fibroblast (CAF). All of these data had a purity adjustment process. And log2 RSEM was used for displaying the gene expression levels.

### CCLE

Cancer Cell Line Encyclopedia (CCLE) database can be used to explore gene variants, candidate targets, small molecules and biotherapeutics, and characterize cancer cell lines [60]. In this study, the mRNA expression level of HCC cell lines was obtained from the CCLE dataset (https://portals.broadinstitute.org/ccle).

### Cell culture

Hep3B cells were purchased from Zhong Qiao Xin Zhou Biotechnology Co., Ltd. (Shanghai, China) and were cultured in MEM medium (Gibco, USA) containing 10% fetal bovine serum (FBS) (Gibco, USA), 100 μg/mL streptomycin and 100 IU/mL penicillin (Gibco, USA). They were cultured at 37°C, 5% CO_2_.

### Lentiviral transduction

Stable KD of IGF2BP3 was obtained by a lentiviral-based shRNA system. Target specific shRNAs or nontarget control (NC) were subcloned into GV493(hU6-MCS-CBh-gcGFP-IRES-puromycin) vector. To isolate infected Hep3B cells, Puromycin selection was performed at 2 ug/ml for 7 days. The KD efficiencies were verified at both mRNA and protein levels. The shRNA sequences used in this study are as follows. shIGF2BP3#1, 5′-ATAGGTTACATTTACAACTGC-3′, shIGF2BP3#2, 5′-TAATCCAGGAATTAAATGTGC-3′, shNC, 5′-TTCTCCGAACGTGTCACGT-3′. 1 μg/ml puromycin for selection was added to the medium of cells transduced with lentivirus.

### RT-PCR

Total RNA was extracted from Hep3B cells which transfected with shNC and shIGF2BP3 lentivirus, according to the RNA Isolater (Vazyme, China). The concentration and purity of total RNA were measured. Through SureScript First-strand cDNA synthesis kit (GeneCopoeia, USA), total RNA was used to obtain cDNA by reverse transcription reaction. The RT-PCR assays were carried out by a protocol from Power SYBR Green (Takara, Hangzhou, Zhejiang, China). The relative expressions of genes were calculated and normalized using the 2^−ΔΔCt^ methods relative to GAPDH. Specific primer sequences were as follows: IGF2BP3: 5′-ACGAAATATCCCGCCTCATTTAC-3′ (forward), 5′-GCAGTTTCCGAGTCAGTGTTCA-3′ (reverse); GAPDH: 5′-CTGGGCTACACTGAGCACC-3′ (forward), 5′-AAGTGGTCGTTGAGGGCAATG-3′ (reverse).

### Western blot

Whole cell lysates were collected in RIPA lysis buffer (Beyotime, China). The antibodies used in this study are IGF2BP3 (#ab179807, Abcam), and β-actin (#20536-1-AP, Proteintech) antibodies. HRP-conjugated anti-rabbit secondary antibody was used for the ECL detection.

### Migration assay

800 μl of conditioned medium containing 20% fetal bovine serum was used as a chemotactic agent for the bottom chamber of the 24-well plate. Serum-free medium of cells transfected with lentivirus shNC and shIGF2BP3 were prepared as suspension at 5 × 10^5^ cells/ml and 200 μl of the suspension was inoculated in transwell chambers. After 48 hrs of incubation, the cells were fixed in methanol for 30 mins and stained with crystal violet for 30 mins.

### Sphere formation assay

Lentiviral transfected shNC and shIGF2BP3 cells were digested with trypsin into single cell suspensions and seeded into special 24-well plates (with 1% polyHEMA (Sigma)-coated) at a density of 3000 cells per well. Cells were grown for 10–14 days in special medium (which including serum-free DMEM/F12 medium (Gibco, USA) supplemented with 4mg/mL insulin (Sigma-Aldrich, St. Louis, MO, USA), B27 (Gemini, USA), 20 ng/mL EGF (MCE, USA), and 20 ng/mL FGF (MCE, USA). The numbers of tumor spheres larger than 50 μm in diameter were calculated.

### Statistical analysis

The differences between two groups were analyzed by Student’s *t*-tests, while differences among more than two groups were analyzed by ANOVA. The two patient cohorts are compared by a Kaplan-Meier survival plot, and the hazard ratio with 95% confidence intervals and log-rank *P* value are calculated. Pearson and Spearman correlation analysis were used to analyze correlations of the data. The *P* < 0.05 was considered as statistical significance.

All methods were performed in accordance with the relevant guidelines and regulations.

### Availability of data and materials

All data generated or analyzed during this study are included in this published article.

## Supplementary Materials

Supplementary Figures

Supplementary Table 1
