# Supplementary material for: The comprehensive expression and functional analysis of m6A modification “readers” in hepatocellular carcinoma
Source: Aging (Albany NY). 2022 Aug 12;14(15):6269–98. doi: 10.18632/aging.204217 (PMC9417225; doi:10.18632/aging.204217)
Supplement: Supplementary Figures [file aging-14-204217-s001.pdf]

SUPPLEMENTARY FIGURES

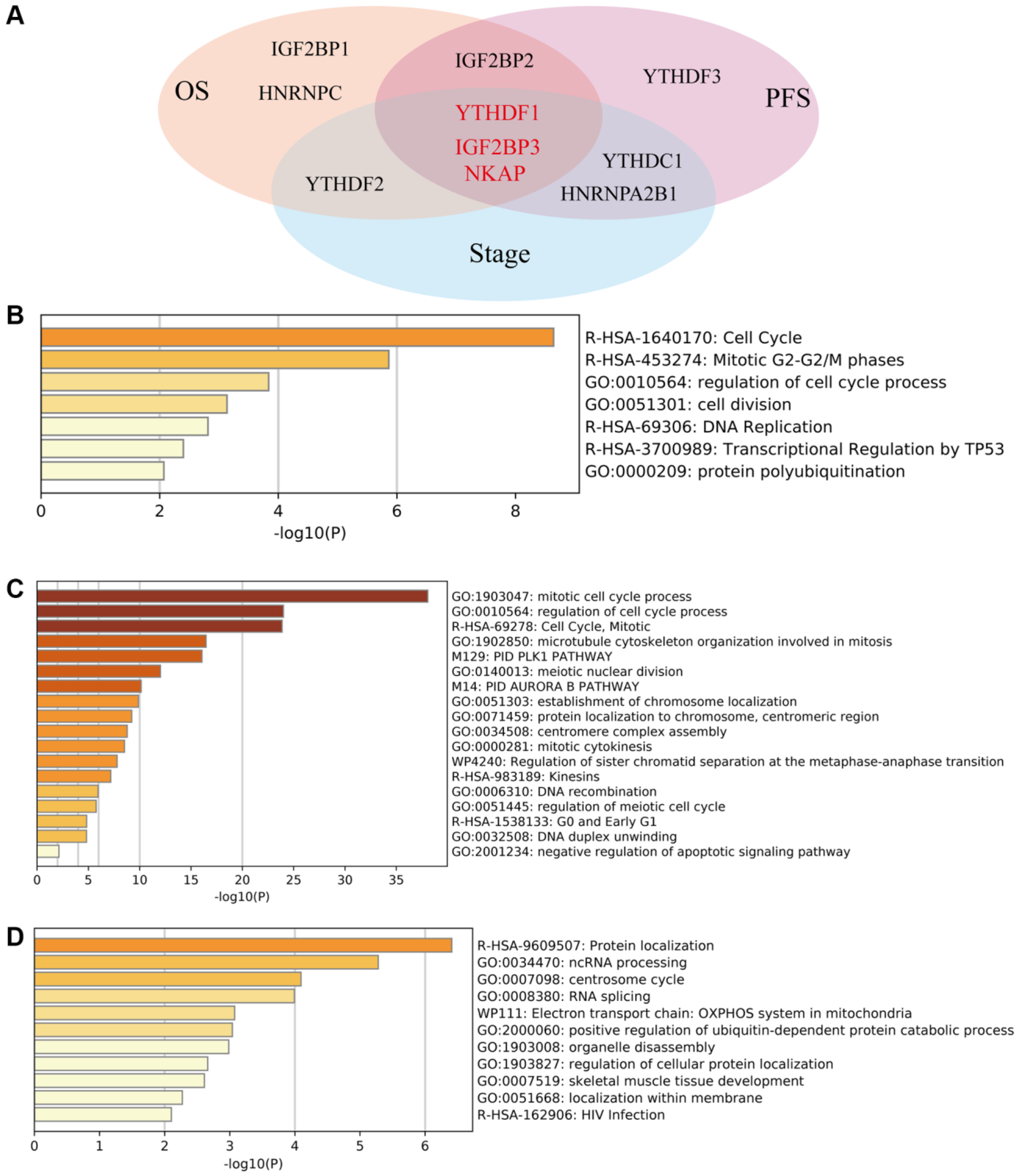

Supplementary Figure 1. Functional enrichment analysis of some key prognosis-related m6A "readers" (YTHDF1, IGF2BP3 and NKAP) in HCC. (**A**) YTHDF1, IGF2BP3 and NKAP were involved in the OS, PFS and stag of HCC based on data from the Kaplan-Meier plotter database, UALCAN database and GEPIA2 database. (**B**) The functional enrichment of the top 20 co-expressed genes of YTHDF1 was analyzed by Metascape. (**C**) The functional enrichment of the top 20 co-expressed genes of IGF2BP3 was analyzed by Metascape. (**D**) The functional enrichment of the top 20 co-expressed genes of NKAP was analyzed by Metascape.

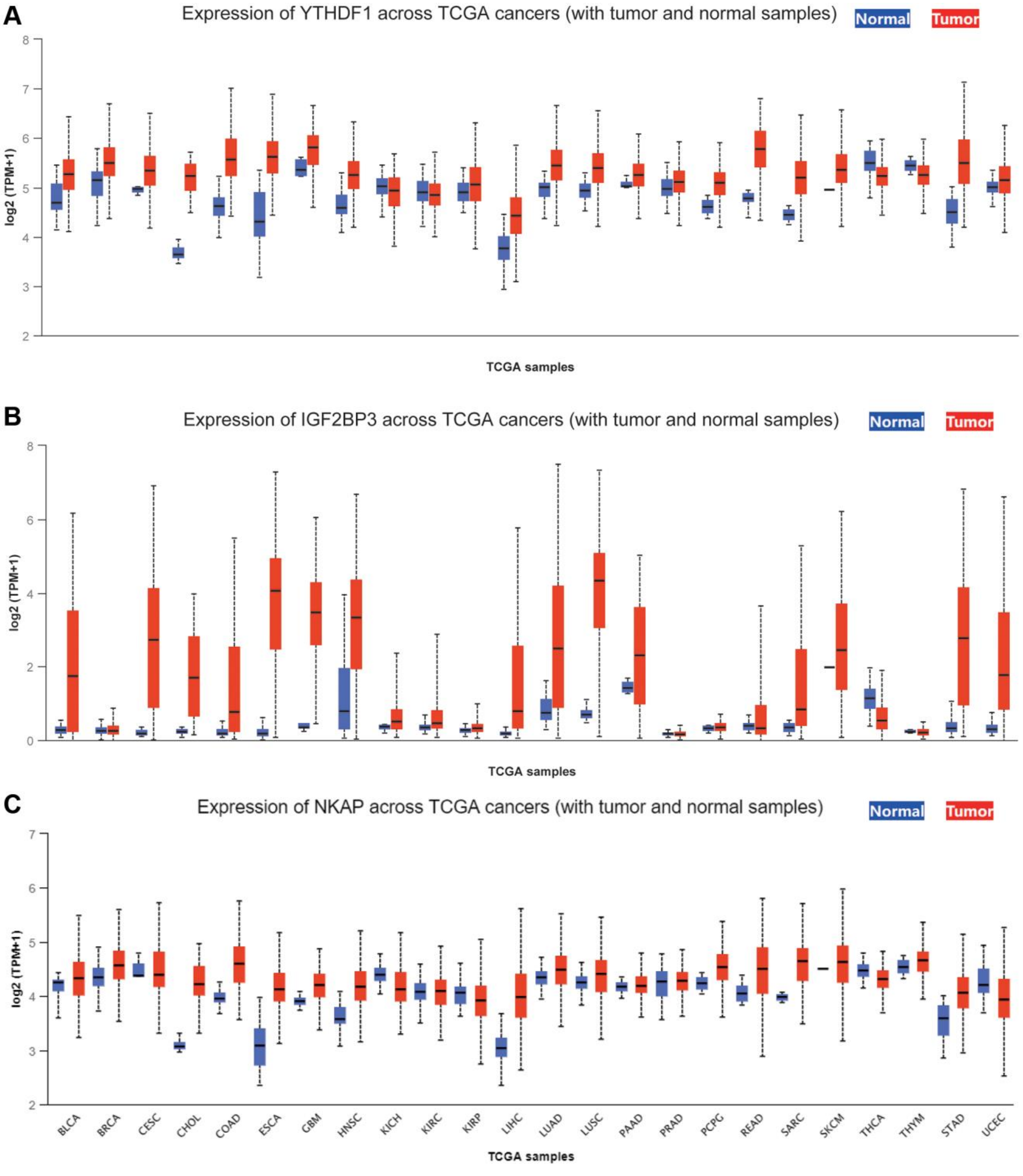

Supplementary Figure 2. The expression levels of some key m6A "readers" (YTHDF1, IGF2BP3 and NKAP) in pan-cancer. (**A**) Expression levels of YTHDF1 in pan-cancerous and normal tissues. (**B**) Expression levels of IGF2BP3 in pan-cancerous and normal tissues. (**C**) Expression levels of NKAP in pan-cancerous and normal tissues.

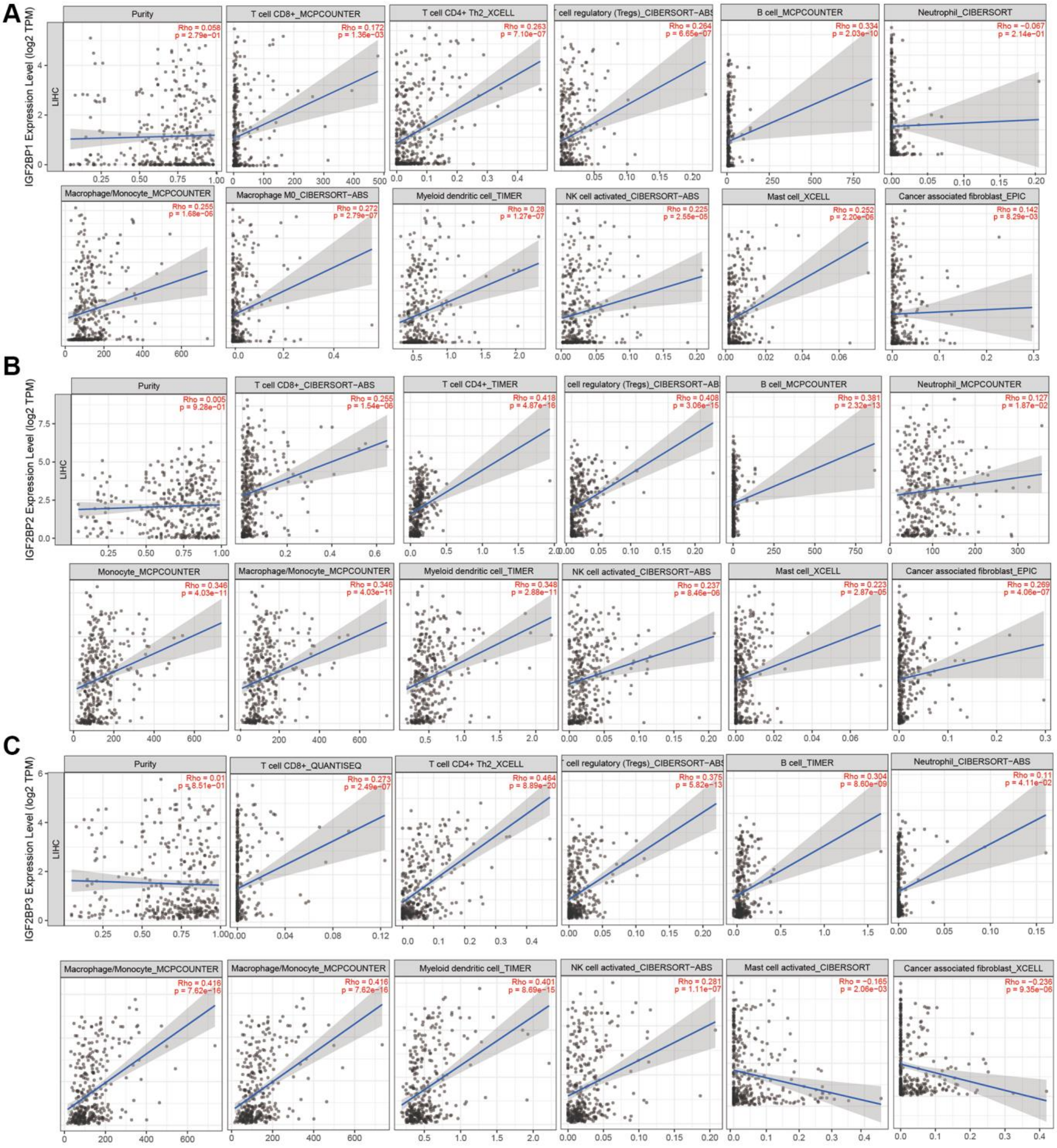

Supplementary Figure 3. Immune cell infiltration of the m6A "readers" (IGF2BP1, IGF2BP2, IGF2BP3) in HCC. (**A**) All of these immune cells had a positive correlation with IGF2BP1, except for the absence of a statistically significant correlation between neutrophil and IGF2BP1. (**B**) All these immune cells we researched had positive correlations with IGF2BP2. (**C**) All these immune cells we researched had positive correlations with IGF2BP3, except for mast cell and CAF. (The mast cell and CAF were negatively related to IGF2BP3). These data were collected from TIMER 2.0 database.

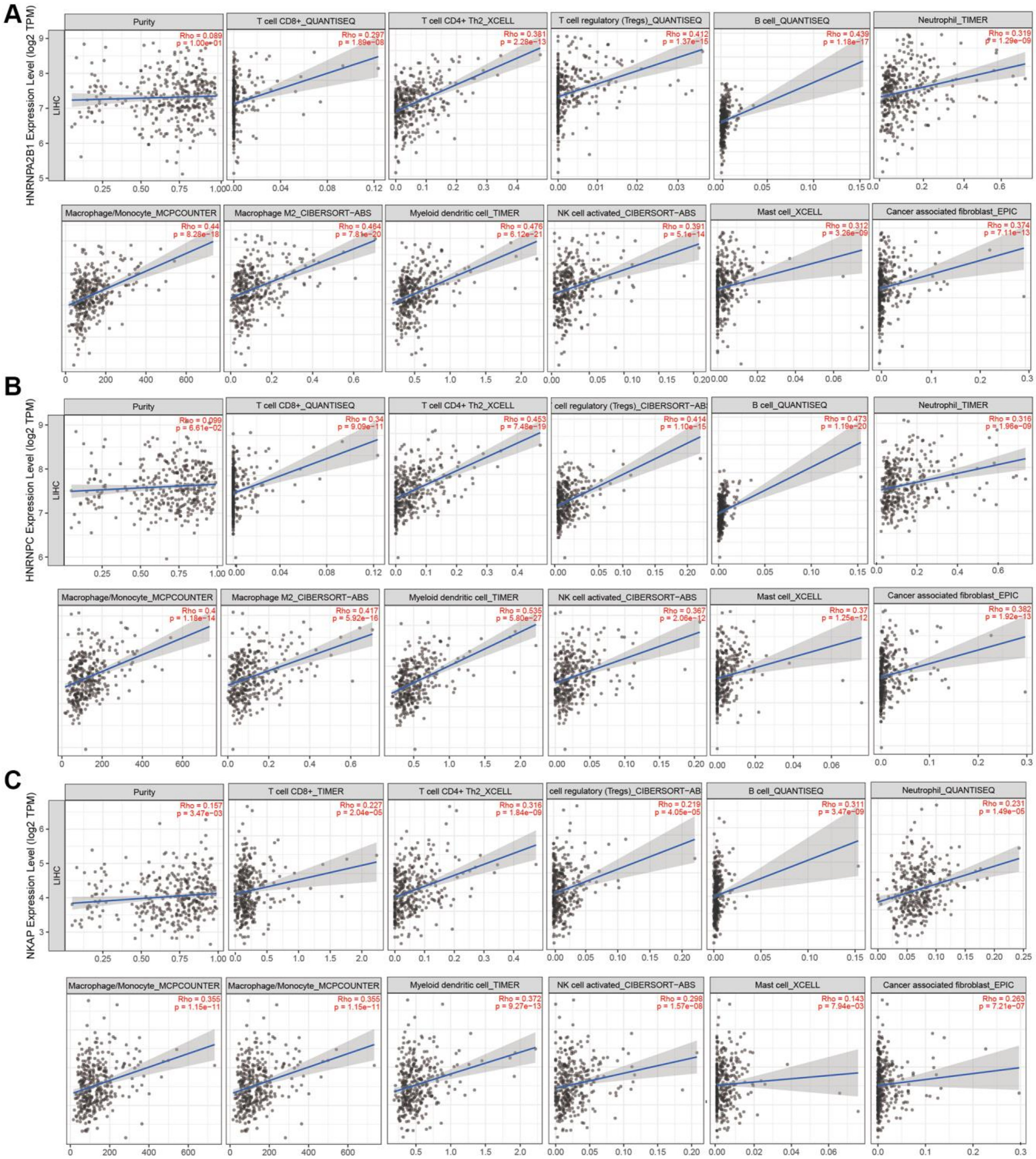

Supplementary Figure 4. Immune cell infiltration of the m6A "readers" (HNRNPA2B1, HNRNPC, NKAP) in HCC. (**A**) All of these 11 immune cells had a positive correlation with HNRNPA2B1. (**B**) All of these 11 immune cells had a positive correlation with HNRNPC. (**C**) All of these 11 immune cells had a positive correlation with NKAP. These data were collected from TIMER 2.0 database.
