# Supplementary material for: The comprehensive expression and functional analysis of m6A modification “readers” in hepatocellular carcinoma
Source: Aging (Albany NY). 2022 Aug 12;14(15):6269–98. doi: 10.18632/aging.204217 (PMC9417225; doi:10.18632/aging.204217)
Supplement: Supplementary Table 1 [file aging-14-204217-s002.docx]

| **Supplementary Table1.** **Top 20 co-expression genes of m6A “readers”** | | |
| --- | --- | --- |
| m6A reader | Correlated Gene | Spearman's Correlation |
| YTHDC1 | ELF2 | 0.755488854 |
| YTHDC1 | GSTCD | 0.701533879 |
| YTHDC1 | CENPC | 0.692185896 |
| YTHDC1 | NUP54 | 0.664491238 |
| YTHDC1 | TET2 | 0.66208407 |
| YTHDC1 | SMARCAD1 | 0.659046666 |
| YTHDC1 | ANKRD17 | 0.652776899 |
| YTHDC1 | SCAF11 | 0.652232399 |
| YTHDC1 | BOD1L1 | 0.645241861 |
| YTHDC1 | CDKN2AIP | 0.643006247 |
| YTHDC1 | CTCF | 0.640537093 |
| YTHDC1 | EPC2 | 0.6391845 |
| YTHDC1 | POLR2B | 0.637087478 |
| YTHDC1 | RIF1 | 0.635931347 |
| YTHDC1 | BPTF | 0.635163594 |
| YTHDC1 | UBA6 | 0.631453432 |
| YTHDC1 | KIAA2026 | 0.631260015 |
| YTHDC1 | KLHL9 | 0.630196221 |
| YTHDC1 | PPP1R12A | 0.629433869 |
| YTHDC1 | GTF3C4 | 0.626600514 |
| YTHDC2 | DMXL1 | 0.785950509 |
| YTHDC2 | APC | 0.69807586 |
| YTHDC2 | PPIP5K2 | 0.692500971 |
| YTHDC2 | PJA2 | 0.684225959 |
| YTHDC2 | BDP1 | 0.668441372 |
| YTHDC2 | RAPGEF6 | 0.659695162 |
| YTHDC2 | RBM27 | 0.655244768 |
| YTHDC2 | SLC25A46 | 0.654979591 |
| YTHDC2 | TTC37 | 0.649589374 |
| YTHDC2 | POLK | 0.649496009 |
| YTHDC2 | C5ORF24 | 0.644677042 |
| YTHDC2 | ERBIN | 0.639611674 |
| YTHDC2 | WDR36 | 0.635418483 |
| YTHDC2 | TMEM161B | 0.635395376 |
| YTHDC2 | FCHO2 | 0.626960085 |
| YTHDC2 | CREBRF | 0.626882923 |
| YTHDC2 | SEC24A | 0.625721906 |
| YTHDC2 | CSNK1G3 | 0.621991939 |
| YTHDC2 | PGGT1B | 0.620839667 |
| YTHDC2 | CHD1 | 0.617480588 |
| YTHDF1 | GMEB2 | 0.570116539 |
| YTHDF1 | TPD52L2 | 0.550069574 |
| YTHDF1 | ARFGAP1 | 0.531783716 |
| YTHDF1 | ZSWIM1 | 0.531157905 |
| YTHDF1 | RAE1 | 0.529994316 |
| YTHDF1 | MRGBP | 0.525369537 |
| YTHDF1 | NCOA5 | 0.51813157 |
| YTHDF1 | AAR2 | 0.486711317 |
| YTHDF1 | PRELID3B | 0.475083655 |
| YTHDF1 | TAF4 | 0.473774232 |
| YTHDF1 | NDRG3 | 0.46742336 |
| YTHDF1 | CEP250 | 0.465072776 |
| YTHDF1 | RALGAPB | 0.462670237 |
| YTHDF1 | SPATA2 | 0.460975008 |
| YTHDF1 | CABLES2 | 0.458953644 |
| YTHDF1 | CYB5A | -0.458605648 |
| YTHDF1 | LSM14B | 0.457614899 |
| YTHDF1 | MYBL2 | 0.456768185 |
| YTHDF1 | TICRR | 0.452598142 |
| YTHDF1 | DNAJC5 | 0.450169883 |
| YTHDF2 | PPP1R8 | 0.666388372 |
| YTHDF2 | KPNA6 | 0.586804682 |
| YTHDF2 | CDC42 | 0.586243721 |
| YTHDF2 | UTP11 | 0.577849881 |
| YTHDF2 | TAF12 | 0.545813625 |
| YTHDF2 | ZCCHC17 | 0.543312577 |
| YTHDF2 | USP48 | 0.528589984 |
| YTHDF2 | PTP4A2 | 0.527719092 |
| YTHDF2 | ELOA | 0.522971113 |
| YTHDF2 | FBXO42 | 0.520978814 |
| YTHDF2 | TXLNA | 0.520164765 |
| YTHDF2 | TMEM50A | 0.512910593 |
| YTHDF2 | STX12 | 0.510425268 |
| YTHDF2 | DNAJC8 | 0.507034777 |
| YTHDF2 | EMC1 | 0.495097184 |
| YTHDF2 | SZRD1 | 0.489515094 |
| YTHDF2 | EIF4G3 | 0.483791799 |
| YTHDF2 | RPA2 | 0.480661631 |
| YTHDF2 | DDOST | 0.476510107 |
| YTHDF2 | GPN2 | 0.475411106 |
| YTHDF3 | VCPIP1 | 0.852691765 |
| YTHDF3 | BPNT2 | 0.738082084 |
| YTHDF3 | ARMC1 | 0.71425011 |
| YTHDF3 | CPNE3 | 0.693660445 |
| YTHDF3 | PCMTD1 | 0.690545452 |
| YTHDF3 | ARFGEF1 | 0.687583238 |
| YTHDF3 | LYPLA1 | 0.680121503 |
| YTHDF3 | UBE2W | 0.675688598 |
| YTHDF3 | EFR3A | 0.670014944 |
| YTHDF3 | WWP1 | 0.661405824 |
| YTHDF3 | DPY19L4 | 0.657920714 |
| YTHDF3 | RB1CC1 | 0.647747796 |
| YTHDF3 | TGS1 | 0.647192793 |
| YTHDF3 | RAB2A | 0.637821279 |
| YTHDF3 | OTUD6B | 0.637678274 |
| YTHDF3 | PHF20L1 | 0.623774618 |
| YTHDF3 | VPS13B | 0.622441017 |
| YTHDF3 | STAU2 | 0.615627435 |
| YTHDF3 | MTFR1 | 0.610487992 |
| YTHDF3 | FAM91A1 | 0.607220478 |
| IGF2BP1 | DNMT3A | 0.607314778 |
| IGF2BP1 | ARID3A | 0.577889015 |
| IGF2BP1 | SALL2 | 0.57060082 |
| IGF2BP1 | TTLL4 | 0.558092351 |
| IGF2BP1 | MCCD1 | 0.555996103 |
| IGF2BP1 | CKAP4 | 0.555034513 |
| IGF2BP1 | IGF2BP2 | 0.552819676 |
| IGF2BP1 | CNOT11 | 0.552409098 |
| IGF2BP1 | MAGED1 | 0.545266937 |
| IGF2BP1 | NREP | 0.544508655 |
| IGF2BP1 | ESR1 | -0.542597671 |
| IGF2BP1 | ACP4 | 0.538117731 |
| IGF2BP1 | TRIM71 | 0.531136733 |
| IGF2BP1 | DLK2 | 0.528253804 |
| IGF2BP1 | MACROH2A2 | 0.528034429 |
| IGF2BP1 | TRIM17 | 0.527321116 |
| IGF2BP1 | DUSP9 | 0.523982929 |
| IGF2BP1 | GPC3 | 0.523475729 |
| IGF2BP1 | BEND3 | 0.523386156 |
| IGF2BP1 | MFSD2A | -0.522965452 |
| IGF2BP2 | LRRC1 | 0.656028124 |
| IGF2BP2 | ARID3A | 0.631627728 |
| IGF2BP2 | CNOT11 | 0.605211113 |
| IGF2BP2 | HSD11B1 | -0.604424584 |
| IGF2BP2 | SALL2 | 0.601809891 |
| IGF2BP2 | SALL4 | 0.597508245 |
| IGF2BP2 | BEND3 | 0.590522984 |
| IGF2BP2 | NREP | 0.587523604 |
| IGF2BP2 | MTHFD1 | -0.5867219 |
| IGF2BP2 | HADH | -0.572863125 |
| IGF2BP2 | MTA3 | 0.566699239 |
| IGF2BP2 | P2RX4 | 0.564659357 |
| IGF2BP2 | CNOT6 | 0.5639923 |
| IGF2BP2 | CYP8B1 | -0.56396465 |
| IGF2BP2 | HPD | -0.563057236 |
| IGF2BP2 | ARHGEF2 | 0.5619402 |
| IGF2BP2 | ARF2P | 0.561512856 |
| IGF2BP2 | NAT2 | -0.561429677 |
| IGF2BP2 | COLCA2 | 0.557950559 |
| IGF2BP2 | IGF2BP1 | 0.552819676 |
| IGF2BP3 | MYBL2 | 0.544137943 |
| IGF2BP3 | BLM | 0.534709373 |
| IGF2BP3 | TTK | 0.533456277 |
| IGF2BP3 | CIP2A | 0.53194752 |
| IGF2BP3 | KIF2C | 0.530720307 |
| IGF2BP3 | FANCD2 | 0.528606702 |
| IGF2BP3 | DEPDC1B | 0.528420487 |
| IGF2BP3 | TRIP13 | 0.521027168 |
| IGF2BP3 | ANLN | 0.51867787 |
| IGF2BP3 | TICRR | 0.518525092 |
| IGF2BP3 | GTSE1 | 0.518124625 |
| IGF2BP3 | CYB5D2 | -0.516999874 |
| IGF2BP3 | BUB1B | 0.514518631 |
| IGF2BP3 | KIF4A | 0.513463324 |
| IGF2BP3 | CCNB1 | 0.511175241 |
| IGF2BP3 | MCM10 | 0.509541997 |
| IGF2BP3 | SMARCD1 | 0.507562301 |
| IGF2BP3 | NCAPG | 0.504933937 |
| IGF2BP3 | CDC20 | 0.504768202 |
| IGF2BP3 | CENPO | 0.503254013 |
| HNRNPA2B1 | SRSF1 | 0.593100255 |
| HNRNPA2B1 | HNRNPL | 0.590680226 |
| HNRNPA2B1 | SFPQ | 0.586213371 |
| HNRNPA2B1 | HNRNPU | 0.578814137 |
| HNRNPA2B1 | HNRNPK | 0.569924922 |
| HNRNPA2B1 | UBA2 | 0.563254346 |
| HNRNPA2B1 | HNRNPD | 0.549604807 |
| HNRNPA2B1 | CENPK | 0.547355561 |
| HNRNPA2B1 | HNRNPH3 | 0.542099862 |
| HNRNPA2B1 | ANLN | 0.535515192 |
| HNRNPA2B1 | CPSF6 | 0.533491514 |
| HNRNPA2B1 | NUF2 | 0.532908176 |
| HNRNPA2B1 | HNRNPH1 | 0.532347215 |
| HNRNPA2B1 | RBMX | 0.53224279 |
| HNRNPA2B1 | TCERG1 | 0.531645563 |
| HNRNPA2B1 | KIF18A | 0.529283662 |
| HNRNPA2B1 | KNTC1 | 0.527592291 |
| HNRNPA2B1 | MELK | 0.524782341 |
| HNRNPA2B1 | CENPF | 0.523898075 |
| HNRNPA2B1 | RACGAP1 | 0.522129286 |
| HNRNPC | SNRPD1 | 0.590661965 |
| HNRNPC | HNRNPL | 0.586593004 |
| HNRNPC | HNRNPA1 | 0.574421613 |
| HNRNPC | SRSF3 | 0.57427475 |
| HNRNPC | ERH | 0.572417483 |
| HNRNPC | BRIX1 | 0.563419985 |
| HNRNPC | HNRNPU | 0.560404787 |
| HNRNPC | NOP58 | 0.554962615 |
| HNRNPC | SRSF7 | 0.547798723 |
| HNRNPC | HNRNPA3 | 0.532195464 |
| HNRNPC | ANP32B | 0.530464227 |
| HNRNPC | SNRPF | 0.530110572 |
| HNRNPC | CPED1 | -0.524036194 |
| HNRNPC | SNRPG | 0.521042601 |
| HNRNPC | SNRPE | 0.518377199 |
| HNRNPC | HNRNPCL3 | 0.518279271 |
| HNRNPC | HNRNPD | 0.517445608 |
| HNRNPC | RAN | 0.510555637 |
| HNRNPC | FUS | 0.509913657 |
| HNRNPC | CCDC59 | 0.509374205 |
| NKAP | RNF113A | 0.681811845 |
| NKAP | MCTS1 | 0.661785713 |
| NKAP | LAS1L | 0.646652109 |
| NKAP | EMD | 0.626232713 |
| NKAP | VBP1 | 0.620580149 |
| NKAP | RBMX2 | 0.613174742 |
| NKAP | NAA10 | 0.592706477 |
| NKAP | SLC25A14 | 0.589510207 |
| NKAP | ATP6AP1 | 0.573756228 |
| NKAP | EOLA1 | 0.571443452 |
| NKAP | EOLA2 | 0.564979668 |
| NKAP | HAUS7 | 0.564683627 |
| NKAP | LAGE3 | 0.562408661 |
| NKAP | FAM50A | 0.55677873 |
| NKAP | CCNQ | 0.556359231 |
| NKAP | NSDHL | 0.544486197 |
| NKAP | PIN4 | 0.541353971 |
| NKAP | PDZD11 | 0.537390206 |
| NKAP | FAM3A | 0.521336842 |
| NKAP | PIN4P1 | 0.520094805 |
